# Littoral macroinvertebrate communities of alpine lakes along an elevational gradient (Hohe Tauern National Park, Austria)

**DOI:** 10.1371/journal.pone.0255619

**Published:** 2021-11-29

**Authors:** Anne Bartels, Ulrike G. Berninger, Florian Hohenberger, Stephen Wickham, Jana S. Petermann

**Affiliations:** Department of Biosciences, University of Salzburg, Salzburg, Austria; King’s College London, UNITED KINGDOM

## Abstract

Alpine lakes support unique communities which may respond with great sensitivity to climate change. Thus, an understanding of the drivers of the structure of communities inhabiting alpine lakes is important to predict potential changes in the future. To this end, we sampled benthic macroinvertebrate communities and measured environmental variables (water temperature, dissolved oxygen, conductivity, pH, nitrate, turbidity, blue-green algal phycocyanin, chlorophyll-a) as well as structural parameters (habitat type, lake size, maximum depth) in 28 lakes within Hohe Tauern National Park, Austria, between altitudes of 2,000 and 2,700 m a.s.l. The most abundant macroinvertebrate taxa that we found were *Chironomidae* and *Oligochaeta*. Individuals of *Coleoptera*, *Diptera*, *Hemiptera*, *Plecoptera*, *Trichoptera*, *Tricladida*, *Trombidiformes*, *Veneroida* were found across the lakes and determined to family level. *Oligochaeta* were not determined further. Generalized linear modeling and permanova were used to identify the impact of measured parameters on macroinvertebrate communities. We found that where rocky habitats dominated the lake littoral, total macroinvertebrate abundance and family richness were lower while the ratio of *Ephemeroptera*, *Plecoptera* and *Trichoptera* (EPT) was higher. Zoo- and phytoplankton densities were measured in a subset of lakes but were not closely associated with macroinvertebrate abundance or family richness. With increasing elevation, macroinvertebrate abundances in small and medium-sized lakes increased while they decreased in large lakes, with a clear shift in community composition (based on families). Our results show that habitat parameters (lake size, habitat type) have a major influence on benthic macroinvertebrate community structure whereas elevation itself did not show any significant effects on communities. However, even habitat parameters are likely to change under climate change scenarios (e.g. via increased erosion) and this may affect alpine lake macroinvertebrates.

## Introduction

Alpine lakes constitute extreme habitats that support highly specific aquatic communities and are among those habitats impacted the least by human activities [1]. However, their specific water chemistry and environmental setting make them susceptible to subtle changes in climate [2–4]. Alpine lakes may thus be suited to serve as sentinels for climate change [5] and may reveal early stages of changing conditions in mountain areas. Benthic communities in alpine lakes serve as important indicators of global change and several studies have been conducted that deal with macroinvertebrate communities (invertebrates larger than 1 mm such as beetles, insect larvae or molluscs) and the ecological state of alpine lakes in Europe [6–10].

Altitude is a multi-faceted ecological parameter that is closely related to other environmental variables which are often hard to disentangle [11], because with increasing altitude, many environmental parameters change. Mean annual air temperature as well as annual minimum temperature is lower in higher elevations, resulting in a decrease of ice-free days in high-alpine lakes and a more shallow position of the thermocline [12]. Increasing elevation comes with decreasing atmospheric pressure, leading to decreasing dissolved oxygen concentrations, whereas lower water temperature (which can be expected in increasing elevation) leads to higher oxygen saturation. Generally, alpine lakes are characterized by a high oxygen saturation and oligo- or even ultraoligotrophic conditions. Oxygen is especially interesting against the background of climate change, as rising temperatures are expected to result in reduced oxygen availability in aquatic habitats and, as a consequence, in a shift in their macroinvertebrate assemblages [12]. Alpine lakes are set in environments with short vegetation periods, where productivity of plants in the catchment as well as aquatic macrophytes, zoo- and phytoplankton is naturally low. This low production rate leads to oligotrophic or ultraoligotrophic lake conditions and limited food availability for macrozoobenthos unless there is a considerable amount of anthropogenic land use or tourism (e.g. a highly frequented hiking trail that leads right to the lake) within the catchment. While high inputs of nitrate and phosphates may lead to considerable alterations of macroinvertebrate communities in freshwater systems [9,13], nitrate, phosphate and, related to this, chlorophyll-a are usually low in high alpine lakes. Furthermore, although pH values can be low due to atmospheric acidification, if lakes are set in calcareous catchments, acid deposition can be buffered effectively [9].

As environmental parameters change along an altitudinal gradient, benthic macroinvertebrate communities may also change. Previous studies have shown that communities in high-altitude lakes are dominated by insect larvae, particularly *Chironomidae* and *Oligochaeta* [7,9,11]. Cold-stenothermic species (species only thriving in cold temperatures) play an important role as indicators of climate change [7], as these species’ ranges are expected to shift upslope as a result of changing climatic conditions [14,15]. Macroinvertebrate taxa sometimes respond in occurrence rather than abundance along the elevational gradient, meaning that with increasing elevation, the number of taxa (e. g. species richness in diving beetles [16]) may decrease [11]. However, macroinvertebrates do not respond uniformly across taxonomic groups to changes in altitude. Incidence of *Planariidae* (planarians), *Halacaridae* (water mites), *Perlodidae* (stoneflies) and *Diamesinae* (non-biting midges, a subfamily of *Chironomidae*) has been found to be positively related to altitude [11] while species richness of non-insect macroinvertebrate fauna is not affected by increasing elevation [7]. Major changes in community composition have been found between elevations below the tree line and higher altitudes [17]. In contrast, richness may also be highest in intermediate elevations [9]. In the Alps and Pyrenees, there is an indication for an ecological threshold between 2,500 and 2,600 m a.s.l., above which abundances and richness strongly decrease [9,11]. This threshold, however, has not been reported for other European mountain ranges [6,18,19].

Dissolved oxygen is known to be of importance to macroinvertebrates [20], even in high-altitude aquatic systems that are thought to be oxygen saturated [21]. In addition, lake size, pH, ice-cover duration and fine substrate presence have also been found to be among the most influential environmental drivers of macroinvertebrate community composition and distribution in high altitude lakes [11,22]. Habitat types play an important role for community composition, as sandy and rocky littoral have been shown to be preferred by different species of *Chironomidae* [22–24]. It has been demonstrated that environmental parameters change with lake size, depth and catchment area [25], potentially altering the sensitivity of the lakes and their communities to environmental influences and stressors and subsequently, the effect of elevation on benthic macroinvertebrate communities. These interactive responses highlight the need for additional data.

In this study, we sampled 28 high-alpine lakes for benthic macroinvertebrates along with several physical and chemical water parameters. We hypothesized that community structure of high-altitude lake macroinvertebrates changes along the elevational gradient in the following fashion:

Hypothesis 1: Overall benthic macroinvertebrate diversity and abundance will decrease with increasing elevation.
Hypothesis 2: Compositional changes within the macroinvertebrate communities, for example towards a higher ratio of cold-stenothermic groups (e.g. cold-stenothermic *Dytiscidae*), are expected to occur with increasing elevation.
Hypothesis 3: Environmental and structural parameters such as oxygen, habitat type or lake size have additional and interactive effects with elevation.

## Material and methods

### Study sites

**Fig 1:**
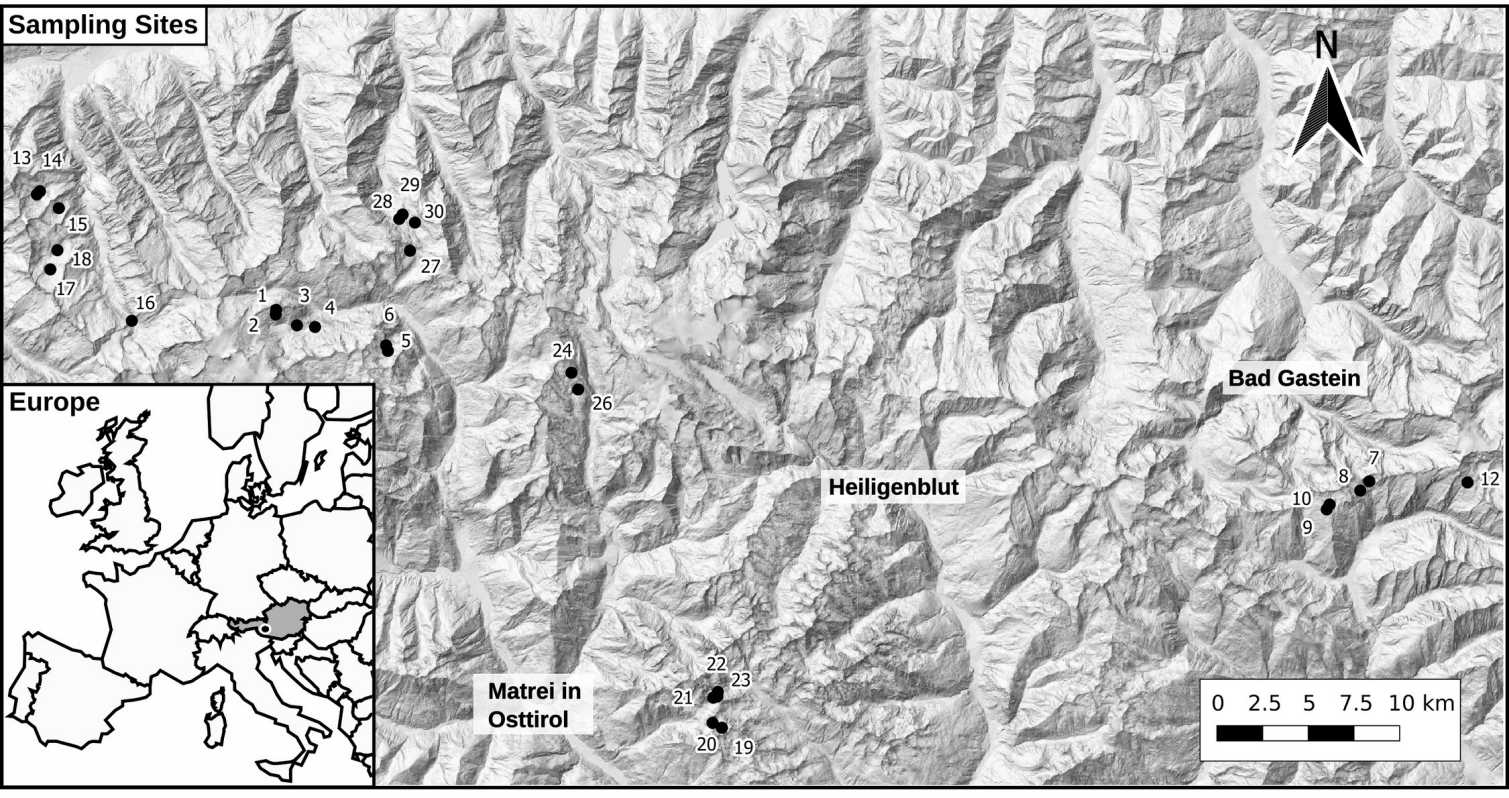
Sampled lakes in Hohe Tauern National Park. The map of Europe in the inset gives the location of Hohe Tauern National Park (black dot) in Austria (grey area) [27].

The Hohe Tauern National Park in Austria (47°04’19.2"N; 12°39’53.7"E), the study site for our investigations, is one of the largest protected areas in central Europe and extends over 1,856 km^2^. Twenty-eight lakes in this National Park between altitudes of 2,083 and 2,727 m a.s.l. were sampled from beginning of July to beginning of August 2018 (see Fig 1 and S1 Table). Field work was permitted by Hohe Tauern Nation Park administration (no field permit number was assigned). Lakes were located in different regions of the National Park or at the National Park border to include a wide range of conditions. Since all lakes are located above the tree line, vegetation in lake catchments was scarce, dominated by lichen and low growing plants and set on gneiss and slate bedrock. The area was taken under protection between 1981 and 1991 (depending on the federal state the respective part of the National Park belongs to). Since then, the main influences have been tourism (as an abundance of hiking trails traverses the area) and alpine farming (extensive cattle and sheep grazing). Lakes were ice-free at the time of sampling. Being set in high alpine environments, almost all lakes were naturally fish-free. Lakes number 5, 6, 21 and 28 were known to have fish due to stocking (primarily brook char, *Salvelinus alpinus*, and brown trout, *Salmo trutta fario* [26]). Lakes number 3 and 4 harbored tadpoles and are subsequently used for spawning by amphibians.

### Measurement of environmental parameters

We followed the guidelines developed for standardized sampling of macroinvertebrates in lakes by Brauns et al. [28]. Habitat types were classified into sediment (maximum grain size of 2 mm), small rocks (up to 20 cm x 15 cm x 5 cm), and large boulders/sheer rock faces. The extent of these three habitat types in the littoral was then estimated. For further analysis, the extent of rocky habitats was calculated as the sum of areas covered by small rocks and boulders/sheer rock faces. Lake size was determined by aerial photograph in Google Earth Pro [29]. To do so, the outlines of the lakes were traced, and the area of the polygon then calculated. Physical and chemical water parameters were measured with a multi-parameter sonde (EXO2 YSI) (see S2 Table) (for lakes 1–18 from a boat, otherwise from a rock or by wading into the lake): water temperature (°C), dissolved oxygen (% saturation), conductivity (μS/cm), pH, nitrate (mg/l), turbidity (FNU), blue-green algae phycocyanin (μg/l) and chlorophyll-a (μg/l). The sonde measured every 10 seconds while being lowered through the water column, thus also determining the position of the thermocline. For further analysis, sonde data from the surface to 1.2 m depth was used because this depth is most relevant for macroinvertebrates [28]. Maximum depth (m) was measured with a sonar by rowing up to 10 transects across lakes. Maximum depth was not measured for lakes 19–28. Sonde measurements at lake 22 were deficient which led to their exclusion in the regression models. Two data loggers had been planted per lake in lakes 1–18 in the previous year and were recovered in 2018. Data loggers measured water temperature at about half a meter depth in six-hour intervals over an entire year. Temperature data for lakes 2, 4 and 16 were not available due to missing or defective loggers. Ice-free days were deduced from available logger data, assuming an ice-cover at water temperatures below 2°C (daily maximum temperature). Additionally, zoo- and phytoplankton samples were taken from the first 18 lakes. Zooplankton was sampled with vertical tows from the hypolimnion to the surface in deeper lakes, and with oblique tows in shallow lakes using a 29 cm diameter net with a 30 μm mesh size. Samples were then fixed in sucrose-formalin and counted under an Olympus SZX16 stereomicroscope microscope equipped with a 0.7–11.5 zoom objective. Phytoplankton samples from lakes 1–18 were taken with a 1.2 L water sampler from the middle of the epilimnion, and when one was present, also from the deep chlorophyll maximum. Samples were fixed with Lugol’s iodine and counted in sampling chambers with a Nikon TE2000 inverted microscope using a 20x objective.

### Sampling of benthic macroinvertebrates

Macroinvertebrate samples were collected from early July to early August in 2018 the littoral of the lakes. A total area of 1 m^2^ was sampled in each lake, using a hand net with a sharp frame (25 cm in width) and 500 μm mesh size. Mixed samples were taken, covering each habitat type proportional to its extent in the lake (100% corresponding to 1 m^2^). E.g., for a lake with half of its littoral zone consisting of sheer rocks and the other half of sandy substrate, 0.5 m^2^ would be sampled on sheer rock and 0.5 m^2^ on sand. One sampling point was chosen randomly for each habitat type. For habitats covering up to 10% of the lake, a standardized area of 0.1 m^2^ was sampled. In sediment, the uppermost 5 cm of the ground were scooped into the net by sweeping it swiftly through the sediment until the target surface area was covered. When sampling large boulders or rock faces, a metal spatula was used to scrape macroinvertebrates off the respective target surface area and collect them in the net. Macroinvertebrates were brushed off small rocks using a toothbrush over water-filled trays. The dimensions of those small rocks were measured, and total surface area was calculated, assuming a suitable geometric form (ellipsoid or cuboid) until the respective target surface area was reached.

Samples were presorted in the field and preserved in 4% formalin. After 3–4 weeks, all samples were rinsed in tap water and transferred to 70% ethanol for further storage. Identification was performed using a stereomicroscope (OLYMPUS SZX16, 11.2x-184x) to the lowest taxon possible, with assistance of different identification keys [30–37]. *Plecoptera* (stoneflies), *Tricladida* (a group of flatworms) and adult *Coleoptera* (beetles) could be determined to species level (S3 Table). *Hemiptera* (true bugs), *Veneroida* (a group of molluscs) and larval *Coleoptera* were determined to genus level. Identification of other groups was restricted by difficulties due to early larval stages or complex taxa. Consequently, *Diptera* (two-winged flies), *Trichoptera* (caddisflies) and *Trombidiformes* (trombidiform mites) were determined to family and *Oligochaeta* (oligochaete worms) as well as the individual of *Hirundinea* (leeches) were determined to subclass level. No Odonata were found.

### Statistical analysis

Since most groups could not be identified to species level, statistical analysis was done at family level for the majority of macroinvertebrates. The combined abundance data from those groups will be subsequently called “total abundance”. Community analysis did not include *Oligochaeta* and *Hirudinea* to maintain comparability by only analyzing groups determined to the same level (family). Family richness was calculated for groups determined at least to family level. Total abundance, family richness as well as EPT abundance/total abundance were tested as response variables. Separate analyses were done additionally for abundances of *Coleoptera*, *Chironomidae*, *Limnephilidae* and *Oligochaeta* because abundance and occurrence at sampling sites was high enough to allow for separate analyses of these groups.

All explanatory variables were tested for colinearity with Pearson’s product-moment correlation test (see S4 Table). Variables that showed collinearity were excluded from the regression analysis according to their ecological importance. Hence, the explanatory variables conductivity, temperature, turbidity, blue-green algal phycocyanin, latitude and longitude were omitted from further analysis. Maximum depth and number of ice-free days were only available for 17 lakes or less and never showed significant effects in the models (data not shown). Consequently, elevation, lake size, the extent of rocky habitats, dissolved oxygen, nitrate, chlorophyll-a, pH and habitat diversity were fitted as explanatory variables in the models. Habitat diversity was calculated with the Shannon Index for the habitat types, using their relative spatial extent per lake. We also investigated the interaction of two important physical parameters: lake size and elevation. Zooplankton abundance per liter and phytoplankton abundance per liter was also only available for 17 lakes and used for additional testing as explanatory variables of total abundance and family richness.

Abundance, family richness as well as separate analyses of *Chironomidae* abundance and *Limnephilidae* abundance were done using generalized linear models with quasipoisson error distribution and a log link. For oligochaete abundance, a generalized linear model with quasipoisson distribution and a log link was used after removing a single outlier with an exceptionally large number of individuals (2,238 individuals in lake 3). Due to low overall abundances, *Coleoptera* data were transformed into presence-absence data and then analyzed using a generalized linear model with binomial distribution and a logit link. Model fit for generalized linear models was tested with D^2^ (a measure for the proportion of deviance explained by the model, see e.g. [38]).

The effect of the environmental variables on community composition was investigated by calculating Bray-Curtis dissimilarities of abundance data. A permanova (function adonis in the R package vegan) was applied with 9999 permutations. Non-metric multidimensional scaling (NMDS) was used to illustrate the differences in community structure graphically.

All data were analyzed using R, Version 3.4.2 [39] and the package “vegan” [40]. Graphs were created with the “ggplot2” [41] and “faraway” [42] packages.

## Results

Four of the sampled lakes were known to have been stocked with fish, but the others were assumed to be fish-free. Especially for the larger lakes though, this is not entirely certain, with incomplete records and contradictory observations [26]. To ensure that a difference in food web structure did not influence our results, we tested our models again, excluding the fish-stocked lakes. Even though fish presence is known to have an important impact on lake communities [43,44], statistical results for our data remained substantially the same (data not shown) so that we decided to keep those lakes in the data set. Overall, we found that lake size and habitat type were the most important factors impacting macroinvertebrate communities in our study area. Elevation itself showed a significant effect on community composition. However, the effect of lake size on abundance and community composition was influenced by elevation. Specific groups responded to elevation (especially chironomids) or other environmental parameters.

### Community structure: Abundance, family richness and composition

A total of 17,927 macroinvertebrates were collected. Chironomids were the most common and the most abundant taxon (66% of all collected individuals), followed by oligochaetes (28% of individuals) (see S1 and S2 Figs).

**Fig 2:**
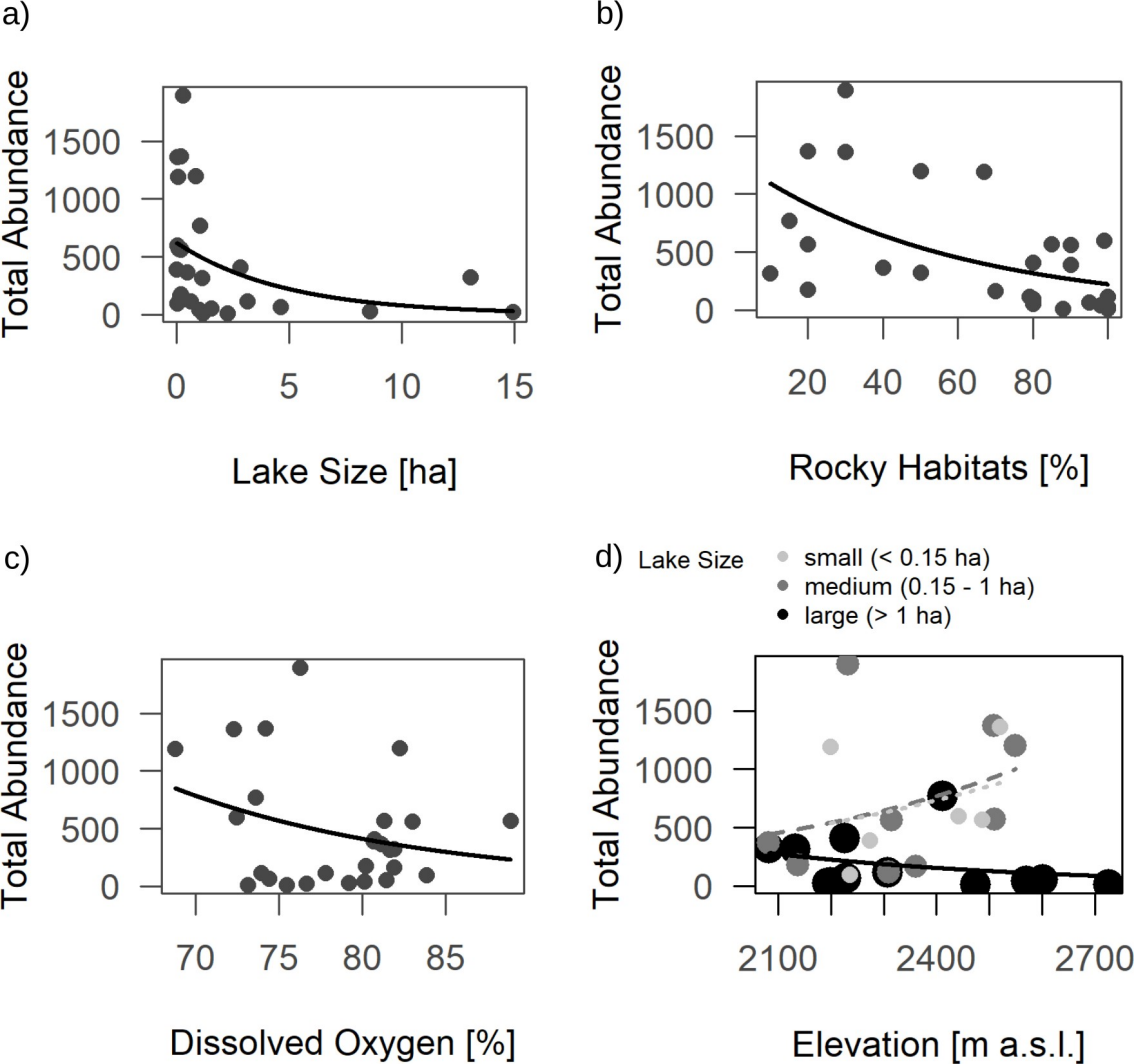
Effect of total macroinvertebrate abundances in alpine lakes and a) lake size; b) proportion of rocky habitats (sum of small rocks and sheer rock faces/boulders); c) dissolved oxygen and d) the interaction of elevation and lake size, where lake size is visualized by varying shades of grey and different regression lines as well as size of the symbols. Regression lines from generalized linear regression with quasipoisson distributions and log-links. All effects shown here are significant (Table 1).

**Fig 3:**
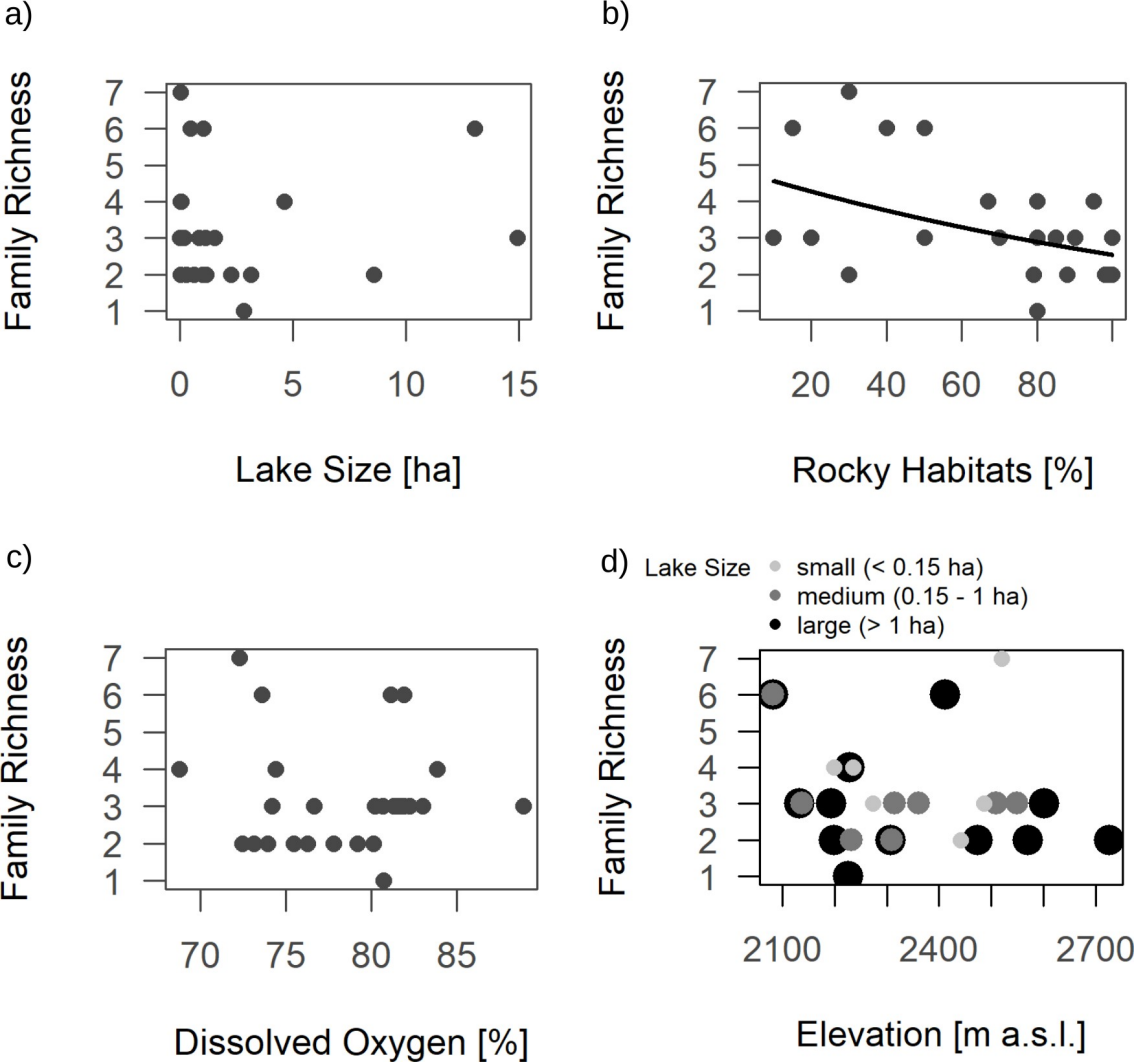
Effect of total family richness in alpine lakes and a) lake size; b) proportion of rocky habitats (sum of small rocks and sheer rock faces/boulders); c) dissolved oxygen and d) the interaction of elevation and lake size, where lake size is visualized by different shades of grey and size of the symbols. Regression lines from generalized linear regression with quasipoisson distributions and log-links are shown for significant effects (Table 1).

**Fig 4:**
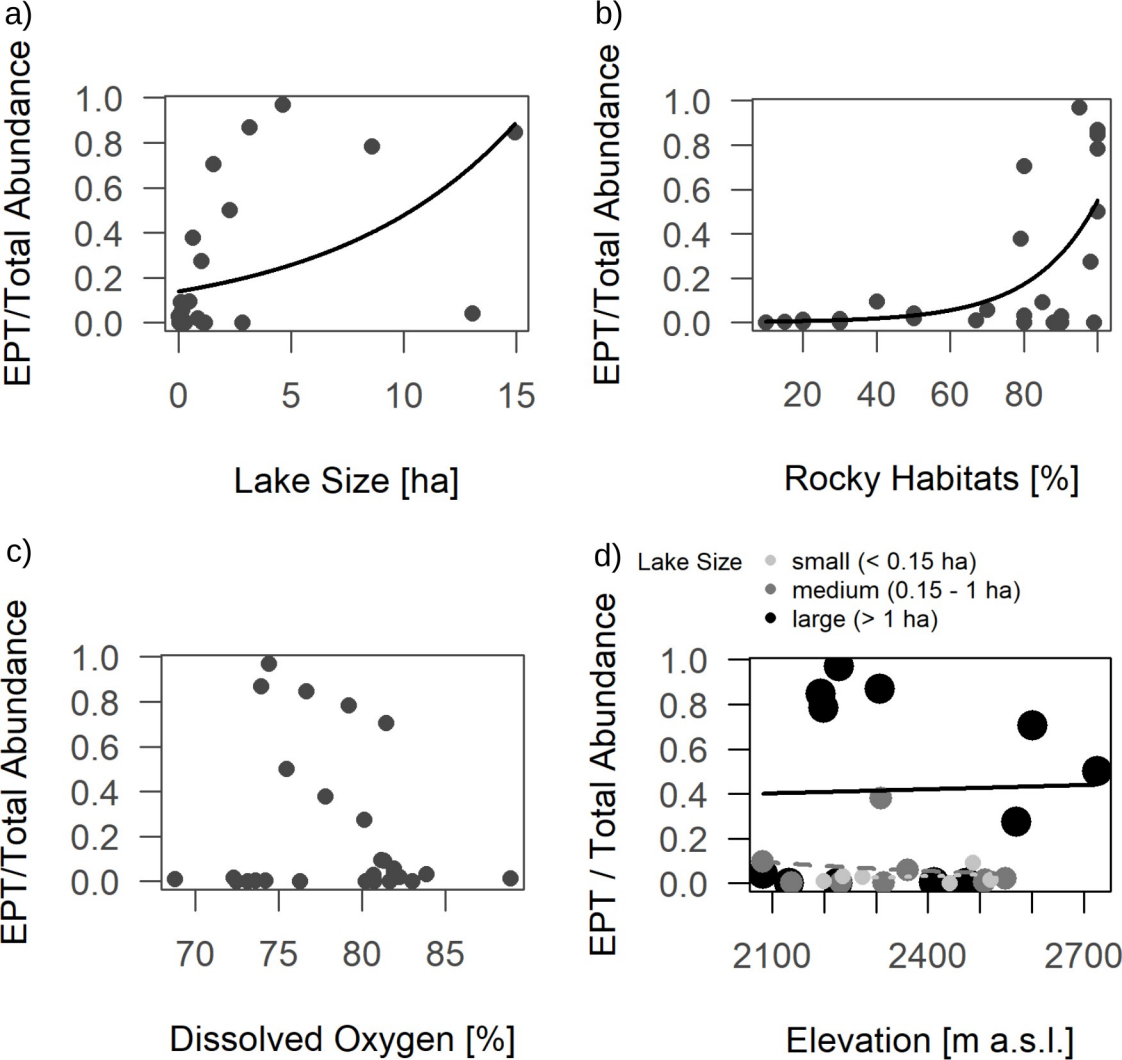
Effect of the ratio of EPT (Ephemeroptera, Plecoptera, Trichoptera) abundance by total abundance in alpine lakes and a) lake size; b) proportion of rocky habitats (sum of small rocks and sheer rock faces/boulders); c) dissolved oxygen and d) the interaction of elevation and lake size, where lake size is visualized by different shades of grey and size of the symbols. Regression lines from generalized linear regression with quasipoisson distributions and log-links are shown for significant effects (Table 1).

Lake size had a negative effect on total abundance (Table 1 and Fig 2A). Abundance was generally lower in lakes with a higher proportion of rocky habitats (Fig 2B). Lakes with 0–33% rocky habitats had mean total macroinvertebrate abundances of 923 individuals (SD = 587), while lakes with 34–66% rocky habitats showed a mean of 627 individuals (SD = 401) and in lakes with more than 66% rocky habitats we collected on average 250 individuals (SD = 306). Contrarily, dissolved oxygen had a significant positive relationship with total macroinvertebrate abundance (Fig 2C). The interaction between elevation and lake size had a strongly significant impact on abundance such that elevation had a positive effect on abundance in small (< 0.15 ha) and medium-sized lakes (0.15–1 ha), while abundance in large lakes (> 1 ha) was generally lower and slightly decreased with increasing elevation (Fig 2D). Family richness was negatively influenced by the share of rocky habitats in the littoral (Table 1, Fig 3). The ratio of EPT (*Ephemeroptera*, *Plecoptera* and *Trichoptera*) abundances by total abundance was significantly influenced by lake size (positive effect, Table 1 & Fig 4A) and showed a highly significant relationship with the share of rocky habitats (positive effect, Table 1 & Fig 4B). The interaction of lake size and elevation has a significant effect on the EPT ratio (Table 1 & Fig 4D). The model was additionally tested with absolute abundance of all EPT-taxa combined (instead of the ratio “Abundance EPT/Total Abundance”), but only the share of rocky habitats showed a tendency for a positive relationship in this case (F_1, 26_ = 3.12, P = 0.095).

**Table 1:** Results of generalized linear modelling (GLM), applying quasipoisson distribution and a log link for total abundance (D^2^ = 0.719), family richness (D^2^ = 0.544) and the ratio of Ephemeroptera, Plecoptera, Trichoptera (EPT) abundance to total abundance (D^2^ = 0.712) of macroinvertebrates in alpine lakes of Hohe Tauern National Park (Austria).

	Total Abundance		Family Richness		EPT per total Abundance	
	F	P	F	P	F	P
Elevation	0.59	0.450	1.53	0.233	0.004	0.950
Lake Size	6.92	**0.018 ↑**	0.22	0.643	7.16	**0.016 ↑**
Rocky Habitats	15.34	**0.001 ↑**	8.68	**0.009 ↑**	29.35	**<0.001 ↑**
Habitat Diversity	1.45	0.245	4.35	0.052	0.03	0.861
Dissolved Oxygen	5.23	**0.035 ↑**	1.82	0.195	0.21	0.649
Nitrate	2.87	0.109	0.001	0.969	4.13	0.058
Chlorophyll-a	0.80	0.385	1.53	0.232	0.64	0.434
pH	2.14	0.162	0.16	0.695	0.44	0.518
Elevation x Lake Size	8.54	**0.010**	0.63	0.444	5.25	**0.0350**

Numerator df = 1 for each explanatory variable, residual degrees of freedom: 26. Significant P-values (P<0.05) are printed in bold. Directions of effects for significant terms are shown by arrows.

Additional testing of the same models (see results above) but with zoo- and phytoplankton abundance per liter as additional explanatory variables in the 17 lakes revealed no effect of phyto- or zooplankton abundance itself on macroinvertebrate abundance or family richness. However, a significant positive effect of elevation (F_1, 16_ = 8.88, P = 0.025, df = 26) and pH on total macroinvertebrate abundance (F_1, 16_ = 6.80, P = 0.040, df = 26) (see S5 Table, S3 Fig) emerged.

**Fig 5:**
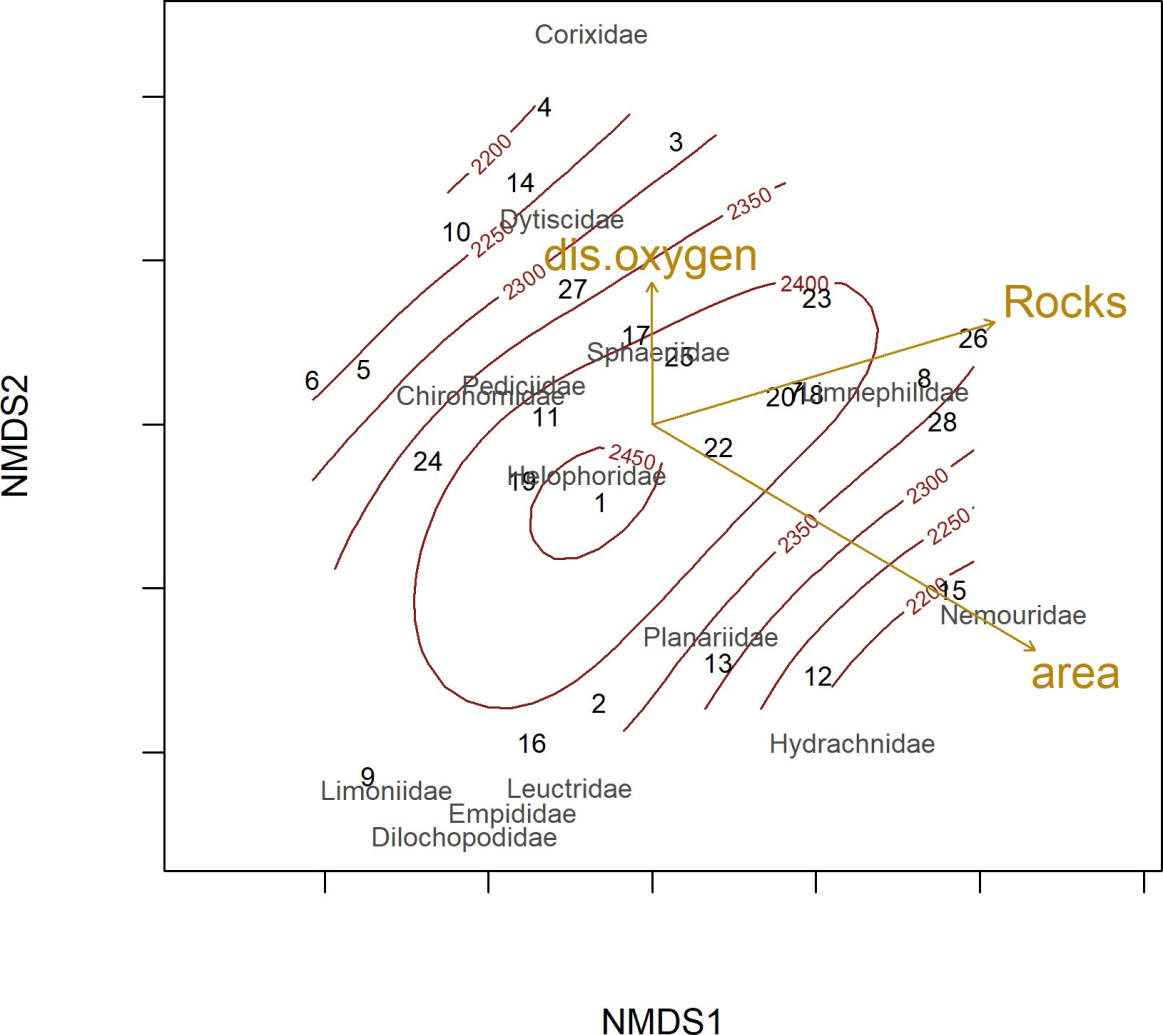
Non-metric multidimensional scaling (NMDS), illustrating similarity in macroinvertebrate community composition of alpine lakes. Dissolved oxygen (“dis.oxygen”), lake size (“area”) and share of rocky habitats (Rocks”) are shown as vectors, elevation shown as contour lines. Community composition was analysed on family level, the taxonomic groups “Hirundinea” and “Oligochaeta” were not included due to low taxonomic resolution. Permanova showed significance effects of elevation (R^2^ = 0.07, P = 0.047), lake size (R^2^ = 0.07, P = 0.040), rocky habitats (R^2^ = 0.15, P = 0.001), dissolved oxygen (R^2^ = 0.067, P = 0.049) and the interaction of elevation and lake size (R^2^ = 0.11, P = 0.005) on community composition, see also Table 2.

In the analysis of community composition, the proportion of rocky habitats and the interaction between elevation and lake size showed highly significant effects (Table 2, Fig 5). Furthermore, elevation, lake size, and dissolved oxygen significantly influenced macroinvertebrate community composition in the studied lakes (Table 2, Fig 5, see also specific groups below).

**Table 2:** Results of permanova, applying Bray-Curtis dissimilarities and running 9,999 permutations to investigate the effect of environmental variables on macroinvertebrate community composition in alpine lakes of Hohe Tauern National Park (Austria).

	Community composition	
	R^2^	P
Elevation	0.071	**0.048**
Lake Size	0.068	**0.040**
Rocky Habitats	0.150	**0.002**
Habitat Diversity	0.013	0.751
Dis. Oxygen	0.067	**0.049**
Nitrate	0.040	0.186
Chlorophyll-a	0.023	0.470
pH	0.014	0.708
Elevation:Lake Size	0.114	**0.005**

R^2^ and P-values are given. Significant P-values (P<0.05) are printed in bold.

### Specific groups

Groups with high abundances and a high occurrence in sampling sites were further investigated separately (at least 1% of total community). ***Chironomidae***, the group with the highest abundance across the study, was present in 25 of the 28 lakes and represented 66% of all collected individuals. The proportion of rocky habitats in the littoral had a negative effect on chironomid abundance, as did lake size (S6 Table, Fig a and b in S4 Fig). The interaction of elevation and lake size had a highly significant effect on chironomid abundance as well (S6 Table, Fig c in S4 Fig). Similar to the effect observed in overall abundance, elevation had a positive effect on chironomid abundance in small and medium-sized lakes but showed a negative effect on chironomid abundance in large lakes.

All ***Trichoptera*** in our samples belonged to the family *Limnephilidae*. They were the third most abundant group encountered in sampled lakes: 486 individuals were found at 21 sampling sites and 3% of all individuals belonged to this group. Only a few individuals had already reached final larval stages–most were still too young to be determined to lower taxon levels. In six lakes, trichopteran cases were discovered but no larvae were found. None of the tested variables showed a significant effect on trichopteran abundance (S7 Table).

Two families of ***Coleoptera*** were found in the samples: Dytiscidae and Helophoridae, together making up 1% of individuals collected across sites. With 187 individuals at 12 sampling sites, Dytiscidae were distinctly more common than Helophoridae (9 individuals at 3 sampling sites). Lake size had a highly significant negative effect on Coleoptera occurrence (S6 Table, S5 Fig).

***Oligochaeta***, the second largest group in abundance (28% of all individuals collected), had a similarly high occurrence as chironomids (22 out of 28 lakes). Nitrate had a significant negative impact on oligochaetes (S6 Table, S6 Fig).

## Discussion

### Elevation leads to compositional changes in macroinvertebrate communities and has differing effects, depending on lake size

All lakes were inhabited by benthic invertebrates and the generally high abundances of chironomids and oligochaetes as compared to other taxonomic groups that were found align with results of several studies on high mountain lakes [7,9,18,45]. Increasing elevation is generally reflected in harsher conditions and shorter ice-free periods for lakes. Thus, communities in lakes of higher altitudes may be increasingly dominated by cold-stenothermal species [46]. Elevation did not show any significant effect on family richness in our study. Still, *Hydroporus foveolatus*, a cold-stenothermal species [14] was present in five of the studied lakes. Cold-stenothermal species can only thrive in cold environments and are thought to be important indicators for the expected upslope shift of species’ ranges due to climate change [14] and will thus be important in monitoring the impact of climate change on alpine lakes. Even though elevation did not show any significant main effect on abundance in our 28 lakes, it did show a significant positive effect on total abundance in the subset of 17 lakes for which we had phyto- and zooplankton data. This statistical difference was likely a result of the smaller subset of lakes which covered a smaller altitude range than the full dataset. Our findings comply with other studies that observed an increase in abundance along elevations of up to 2,600 m a.s.l. with a following decrease in higher altitudes [8,9]. Temperatures in lakes above that altitude threshold are thought not to be warm enough to support large communities [9]. In this context, it is important to note that all studied lakes were set in either high or very high elevations (our elevational gradient ranged from 2,083 to 2,727 m a.s.l.). If the range had been extended at the lower end (e. g. starting at 1,000 m), there would be more potential for an elevation effect. However, the focus of our study lay on high elevation lakes as taxa in these are most vulnerable to global warming. Including lakes at or close to valley bottoms would have introduced significantly greater variation into the data set, mostly because of increased anthropogenic impacts on the lakes and catchments due to more intense land use (more grazing, regular fish stocking, more tourism). Lakes higher than 2,727 m a.s.l. were scarce and largely inaccessible in our study area.

Our data revealed an impact of elevation on macroinvertebrate community composition in the sampled lakes. The effects of elevation are expressed by a multitude of factors and are not easily explained [see e.g. 11]. However, the effect of elevation on community composition can be partially explained by availability of food resources. Water temperature affects food availability, as lakes at higher altitudes are characterized by shorter ice-free periods and thus shorter periods that support primary production and food growth as compared to lakes at lower altitudes. It is well known that only few, well-adapted species survive in environments with such restricted time periods of food availability [7,9]. An increase in ice cover duration with increasing altitude may lead to a shift in community composition towards even more specialized species.

Interactive effects of elevation and lake size showed that communities in small (< 0.15 ha) and medium-sized lakes (0.15–1 ha) increased in total abundance with increasing elevation. In large lakes (> 1 ha) they slightly decreased. The opposite trend was expected to occur, as smaller water bodies at high altitudes are exposed to extreme temperature fluctuations throughout the year and will most likely freeze to the bottom during winter or may fall dry during the summer [47,48]. This creates extreme and difficult conditions for macroinvertebrate communities, and we expected lower abundances in smaller lakes with higher elevation. A possible explanation for lower macroinvertebrate abundances in large lakes in our study could be the exposition to wind-induced wave action in large lakes. Exposed lakes at high altitudes experience higher wave action, an effect that is compounded by lake size [49,50]. This may not only reduce abundances in large lakes, but also result in a shift towards more adapted and robust species. A similar trend can be seen in our data set, as abundances seem to strongly decrease in lakes above 2,600 m a.s.l. All lakes above 2,500 m a.s.l. fell in the category of large lakes though, which means that the decrease in abundance at elevations above 2,600 m a.s.l., which has been observed by other authors [8,9], may still occur in small and medium-sized lakes but could not be reflected in our data. However, the observed increase in abundances in small and medium-sized lakes with increasing altitudes may be due to the fact that small water bodies are prone to extreme temperature regimes [47,48], shaping macroinvertebrate communities with robust and well adapted species that thrive in the absence of competitors [51]. Especially the higher number of chironomids in smaller lakes that we observed may contribute to this effect of increasing abundances in smaller lakes. However, our data was not resolved sufficiently to support this notion. With ongoing climate change, ice-free periods in high altitude lakes are expected to increase and abundances in large lakes may rise. The range of cold-stenothermal species will probably be negatively affected. Since our analysis was only done on family level, possible further effects of elevation may have been masked by the taxonomic resolution of our data.

### Effects of environmental parameters

Four of our lakes had a different food web structure than the rest as they were inhabited by fish. Fish presence is known to alter macroinvertebrate communities [43,44] but did not have such fundamental effects on our lake communities. Observed effects in our data set originated from the influence of environmental parameters.

Contrary to Hamerlík et al. [46] who found an increase of chironomid and macroinvertebrate taxa richness with increasing lake area, our results did not show a relationship between family richness and lake size. Chironomid, coleopteran as well as total abundances were lower in larger lakes, which again may be a consequence of higher wave action in larger lakes [49]. Exposure to wind and thus higher water movement in lakes are shaping factors for macroinvertebrate communities [49,52,53] and lead to harsher living conditions. This may have a reducing effect on abundances and lead to the observed community shift with changing lake size. Another reason for this effect may be that seven out of the eight lakes with a size larger than 1.5 ha had a very high proportion of rocky littoral (> 80%). Lakes smaller than 1.5 ha varied more in the amount of rocky littoral. Larger lakes in our samples were thus characterized by harsher habitats with less shelter and less accumulation of fine organic substrate.

The ratio of EPT-species abundance (species of *Ephemeroptera*, *Plecoptera* and *Trichoptera*) per total macroinvertebrate abundance was positively affected by lake size. EPT species did not seem to be negatively affected by increasing lake size or stronger wave action, as they are generally more mobile than chironomids and are able to seek cover in periods of strong winds or, in the case of case-bearing caddisflies, carry their refuge with them. This should be true for coleopterans as well, but we observed the opposite trend for this group: Coleopteran occurrence decreased with increasing lake size. We do not know why coleopterans react differently to lake size. However, this relationship should be viewed with caution since analysis on coleopterans was done on presence/absence rather than abundances. Increasing numbers of EPT species with increasing lake size is a known phenomenon [45]. The observed effect on EPT/total abundance ratio may partially be explained by the significant decrease of chironomid abundances and thus, lower food competition.

The number of rocky habitats played a vital role in determining abundances, family richness and community composition of the studied lakes. A difference in community composition between habitat types was observed in our data, which is in accordance with other studies [24,54]. In small, oligotrophic lakes, habitat structure may be more important in shaping macroinvertebrate communities than water chemistry [54]. Given the high proportion of chironomids, it is not surprising that chironomid abundances drove the effects of most variables on total abundance. The higher the share of rocky habitats was in our study sites, the lower total abundances and chironomid abundances were. Research on chironomid communities in alpine lakes has shown that rocks support the lowest taxa richness while sediment has higher richness [24], which complies with our results of decreasing overall family richness with increasing share of rocky habitats in the littoral.

Differences in total abundance in our study were largely driven by chironomid habitat preference for sediment. Chironomids found in sandy littoral may be highly specialized to those habitat types. Most rocky habitats consisted of sheer rock faces or large boulders and exposition of these habitats to wind can be considered very high, especially compared to sandy patches that were often nested in between large boulders. Rocky habitats mainly consisted of large boulders and interstitial space was thus very small. This harshness of hard substrates and reduced interstitial space could explain lower abundances and family richness in rocky habitats. Additionally, sandy habitats may be more favorable in terms of nutrient storage, possibly explaining higher abundances and family richness in those sedimented areas. On the contrary, the EPT ratio showed a strongly significant, positive relationship to the amount of rocky littoral, reflecting their preference for coarse substrates [55]. This is due to a combination of decreasing chironomid abundance (boosting the relative abundance of EPT species) and an increase in absolute EPT abundances in rocky habitats. The latter was observed in our data by the tendency of trichopteran and total EPT abundances to increase with increasing share of rocky habitats (no significant effects were observed).

Another group with high abundances, Oligochaeta, was mainly observed in littoral sediment. This coincides with findings of other studies, that report oligochaete preference for sandy habitats [23,56]. Rieradevall et al. [23] mention the preference of *Pisidium* species for fine sediments. In our study, the two lakes with large *Pisidium* abundances indeed showed sediment to be 50% and 90% of total habitat, respectively.

Habitat types are considered to be a major influencing variable on community composition of macroinvertebrates [52]. This is reflected in our results, as community composition significantly changed with changing share of rocky littoral. The number of habitat types has a significant impact on species richness of macroinvertebrates in mountain ponds [57]. This corresponds to the positive trend between family richness and habitat diversity in our data (not significant).

Oligochaete abundance related negatively to nitrate concentrations. Increased nitrate levels may indicate nutrient input by cattle or sheep or atmospheric nitrogen deposition. Livestock or their excrements were observed near the shore of about half of the lakes. Large herbivore presence was tested in an additional model as an explanatory variable, yielding similar statistical results as nitrate. This variable was left out of the final model because presence of cattle or sheep was judged subjectively during field work and is thus not as reliable as in-situ measurements of nitrate. It is unclear why oligochaete abundance decreased with increasing nitrate concentrations. The expected relationship would be an increase of oligochaetes with increasing nutrient input [58]. Considering that there was only a weak effect of nitrate on oligochaetes which was barely significant (p = 0.045, F_1,26_ = 4.71, see S7 Table), we do not want to overinterpret this relationship and will refrain from further discussion at this point.

Similar considerations can be applied to the relation between community composition and dissolved oxygen concentrations in the water. Taking a closer look, no specific group seems to be driving this relationship. Although total abundances decreased with increasing oxygen content, only chironomid and coleopteran abundances displayed a tendency to decrease (but no significant relationship). Oxygen content is known to have an impact on macroinvertebrate communities [59] but under the oligotrophic conditions in the studied lakes, no significant effect of dissolved oxygen was expected and the effect we found was weak (R^2^ = 0.067, P = 0.049). Thus, we will refrain from speculating further. Small, shallow mountain lakes that hold a considerable amount of algae and detritus provide favorable living conditions for water beetles [16]–a circumstance that may explain the positive relationship between coleopteran abundance and chlorophyll-a values of the studied lakes.

## Conclusion

Contrary to our first and second hypotheses, we did not find strong effects of elevation per se on communities and macroinvertebrate groups. Mendoza & Catalan [11], however, have noted that elevation itself is associated with many different variables, which we also saw in our dataset. The second hypothesis could be tested regarding general macroinvertebrate community structure, but no statement can be made about specific effects on stenothermal species, due to low taxonomic resolution of our data. The effect of lake size was dependent on elevation, providing support for our third hypothesis, as small and medium-sized lakes showed increasing abundances with increasing elevation, while macroinvertebrate abundances in large lakes slightly decreased. Lake size and habitat structure seemed to be of great importance for macroinvertebrate communities in high alpine lakes. The most obvious effects were produced by habitat type and thus not related to changes in elevation. Chemical parameters only showed weakly significant effects in our analyses.

Alpine lakes are very sensitive to shifts in climate, especially lakes between 1,500 and 2,000 m a.s.l. are thought to be highly affected [4]. A general upslope movement of species’ ranges is anticipated with climate change [14,15]. Insect fauna can be expected to be most influenced by this effect due to their high dependency on altitudinal levels as opposed to non-insect fauna [7]. Variables that showed great importance in our study were structural in nature (habitat type and lake size) and will not change dramatically with climate change. However, glaciers melting under ongoing climate warming will increase sizes of lakes adjacent to them and create new lakes. There will most likely be a change in the habitat structure of those lakes as well, as increased erosion due to thawing of permafrost and more extreme weather events may lead to a higher proportion of sediment in lake littorals. Thus, while climate change may facilitate upslope movement of many species, our data show that habitat change with increasing altitude is clearly multidimensional. Macroinvertebrate communities in alpine lakes will undoubtedly change with a changing climate, but understanding that change will require a very broad spectrum of parameters to be observed.

## Supporting information

S1 Fig Mean abundances of families/subclasses across the study of macroinvertebrates in alpine lakes of Hohe Tauern National Park. Standard error is given by error bars. Due to large differences in abundance, chironomids and oligochaetes are displayed on a different scale.
(TIF)

S2 Fig Mean abundances of families/subclasses across the study of macroinvertebrates in alpine lakes of Hohe Tauern National Park, grouped by share of rocky habitat (given in percent on top of graphs). Standard error is given by error bars. Due to large differences in abundance, chironomids and oligochaetes are displayed on a different scale. Order names are given below taxa.
(TIF)

S3 Fig Effect of total macroinvertebrate abundances in a subset of alpine lakes (number 1–17) for which zoo- and phytoplankton abundances per liter were available and were used as additional explanatory variables and a) lake size; b) proportion of rocky habitats (sum of small rocks and sheer rock faces/boulders); c) pH and d) elevation. Regression lines are from generalized linear regression with quasipoisson distributions and log-links and only shown for significant relationships (see S5 Table).
(TIF)

S4 Fig The relationship between chironomid abundances in alpine lakes and a) lake size; b) proportion of rocky habitats (sum of small rocks and sheer rock faces/boulders; and c) the interaction of elevation and lake size, where lake size is visualized by different shades of grey and size of the dots. Regression lines are based on generalized linear regression with a quasipoisson distribution and a log-link and are significant (S7 Table).
(TIF)

S5 Fig Generalized linear regression with binomial distribution and logit-link between coleopteran occurrence in alpine lakes and lake size. The effect shown here was significant (S7 Table). 1 = coleopterans were present, 0 = coleopterans were absent.
(TIF)

S6 Fig Generalized linear regression with quasipoisson distribution and log-link between oligochaete abundances and nitrate in alpine lakes. The effect shown here was significant (S7 Table).
(TIF)

S1 Table List of sampled lakes in Hohe Tauern National Park, valleys the lakes are located in, sampling date, elevation in meters above sea level (m a.s.l.), estimated habitat coverage (given in percent) in the littoral of sampled lakes, lake size in ha, maximum depth in meters as well as numbers of ice-free days. Habitat types are sheer rock (large boulders and sheer rock faces), small rocks (up to 20 cm x 15 cm x 5 cm) and sediment. Lakes in quotation marks did not have official names and were named for the convenience of this study by the sampling team.
(PDF)

S2 Table List of sampled lakes in “Hohe Tauern” National Park and the parameters measured for each lake as well as zoo- and phytoplankton abundances per litre. (PDF)

S3 Table List of all orders or subclasses, the lowest taxon they were determined to, total abundances across the study, the number of sampling sites (lakes) they were present in and the number of taxa within each order/subclass that were determined for macroinvertebrates in alpine lakes of Hohe Tauern National Park. (PDF)

S4 Table Table of correlations between measured variables in the lakes of Hohe Tauern National Park according to Pearson’s product-moment correlation test. This correlation test was the basis for choosing parameters for further statistical analysis. Only significant correlations are listed. Parameters printed in bold were included in further statistical analysis.
(PDF)

S5 Table Results of generalized linear modelling (GLM), applying quasipoisson distribution and a log link for total abundance in alpine lakes of Hohe Tauern National Park (Austria) for a subset of lakes (lakes number 1–17) for which zoo- and phytoplankton abundances per liter were available and were used as additional explanatory variables. Numerator df = 1 for each explanatory variable, significant P-values are printed in bold. Residual degrees of freedom: 16.
(PDF)

S6 Table Results of generalized linear modelling (GLM), applying quasipoisson distribution and a log link for trichopteran abundances in alpine lakes of Hohe Tauern National Park (Austria). Numerator df = 1 for each explanatory variable, significant P-values are printed in bold. Residual degrees of freedom: 26.
(PDF)

S7 Table Results of generalized linear modelling (GLM), applying quasipoisson distribution and a log link for Chironomidae abundance and Oligochaeta abundance and applying binomial distribution and a logit link for Coleopteran occurrence in alpine lakes of “Hohe Tauern” National Park (Austria). Numerator df = 1 for each explanatory variable, significant P-values are printed in bold. Residual degrees of freedom: 26.
(PDF)

S1 Data Table of lowest determined taxa and their abundances of all sampled lakes. (XLSX)
