# Supplementary figures and images for: Littoral macroinvertebrate communities of alpine lakes along an elevational gradient (Hohe Tauern National Park, Austria)
Source: PLoS One. 2021 Nov 29;16(11):e0255619. doi: 10.1371/journal.pone.0255619 (PMC8629281; doi:10.1371/journal.pone.0255619)

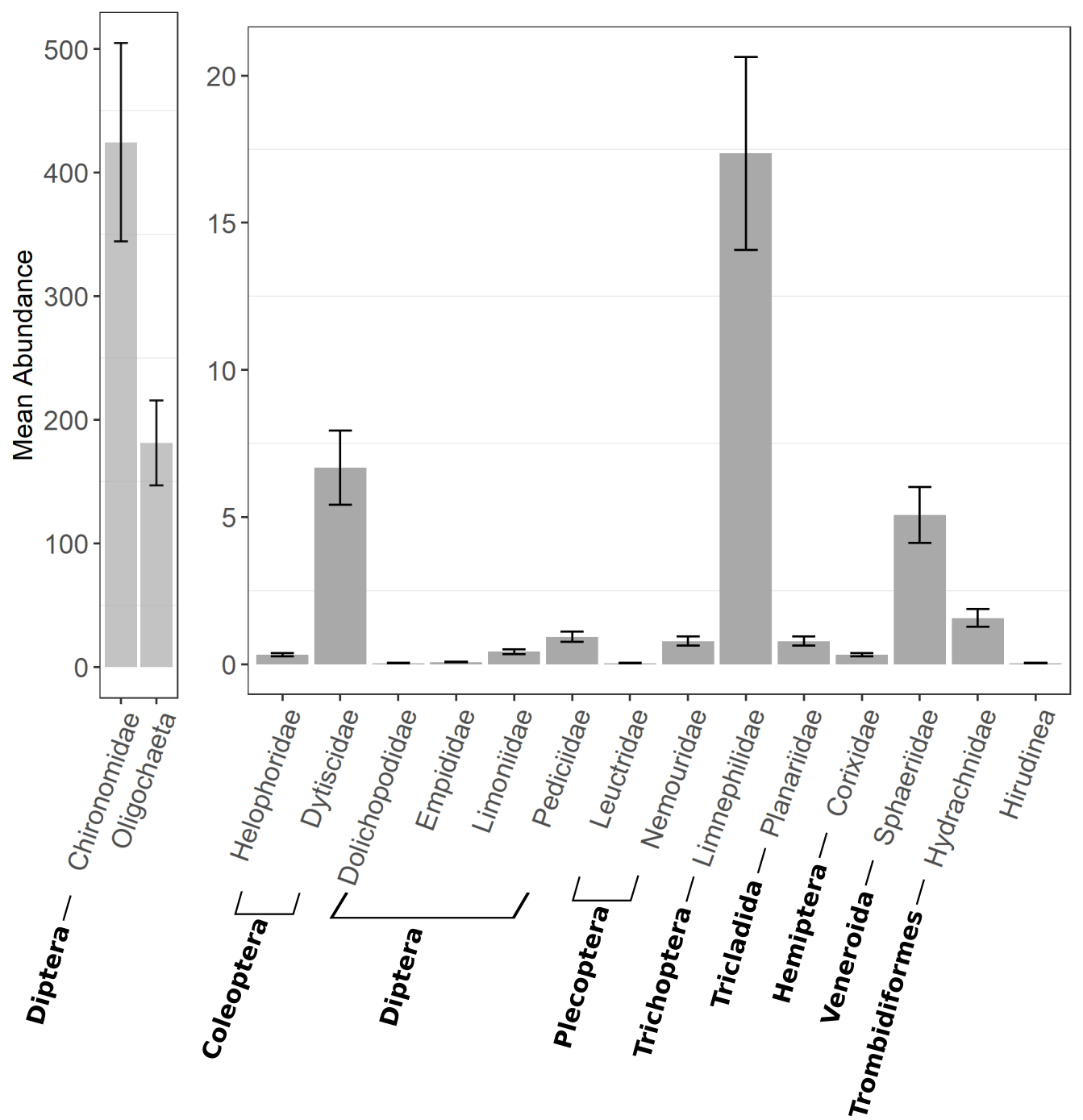

Supplement: S1 Fig — Standard error is given by error bars. Due to large differences in abundance, chironomids and oligochaetes are displayed on a different scale. (TIF) [file pone.0255619.s001.tif]

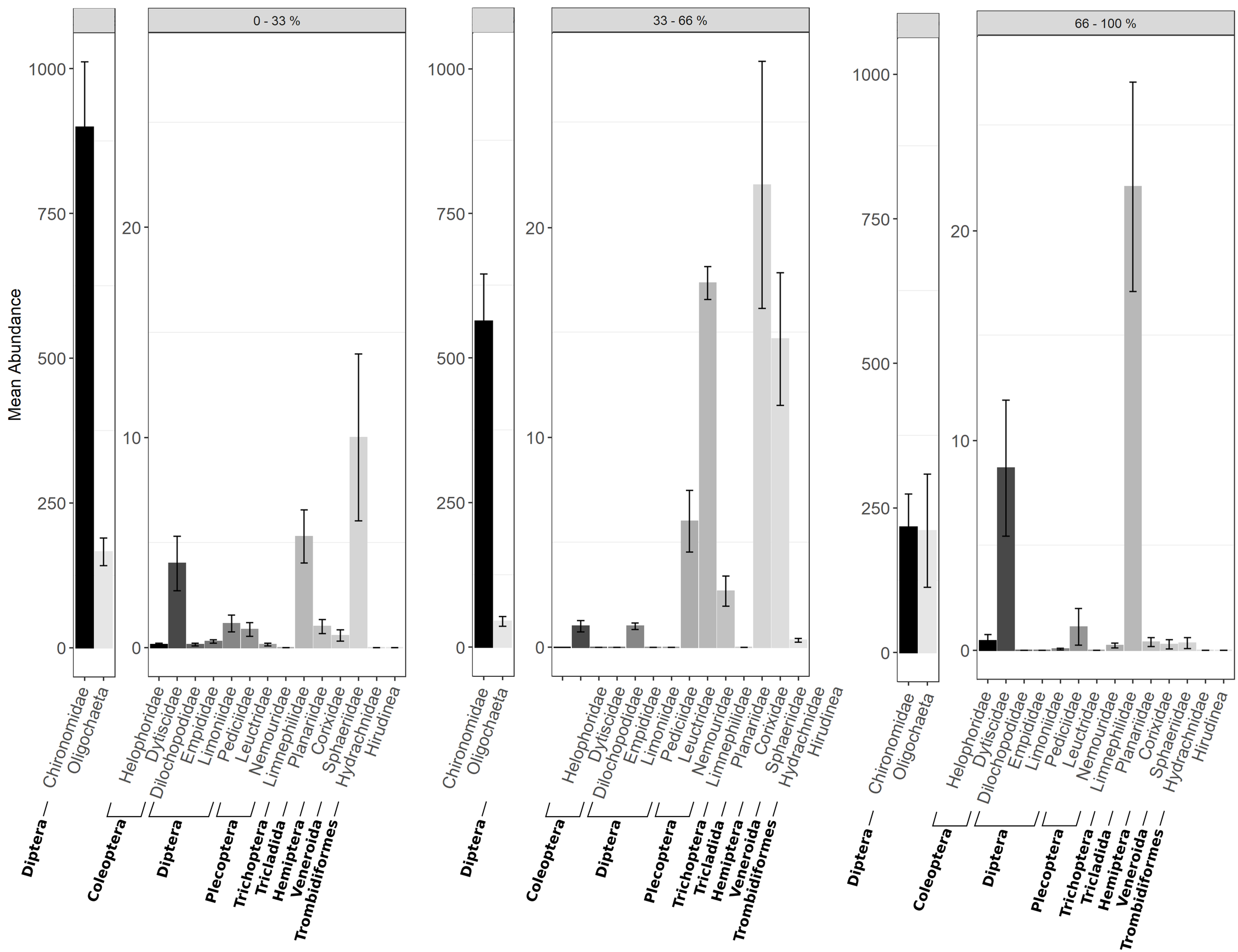

Supplement: S2 Fig — Standard error is given by error bars. Due to large differences in abundance, chironomids and oligochaetes are displayed on a different scale. Order names are given below taxa. (TIF) [file pone.0255619.s002.tif]

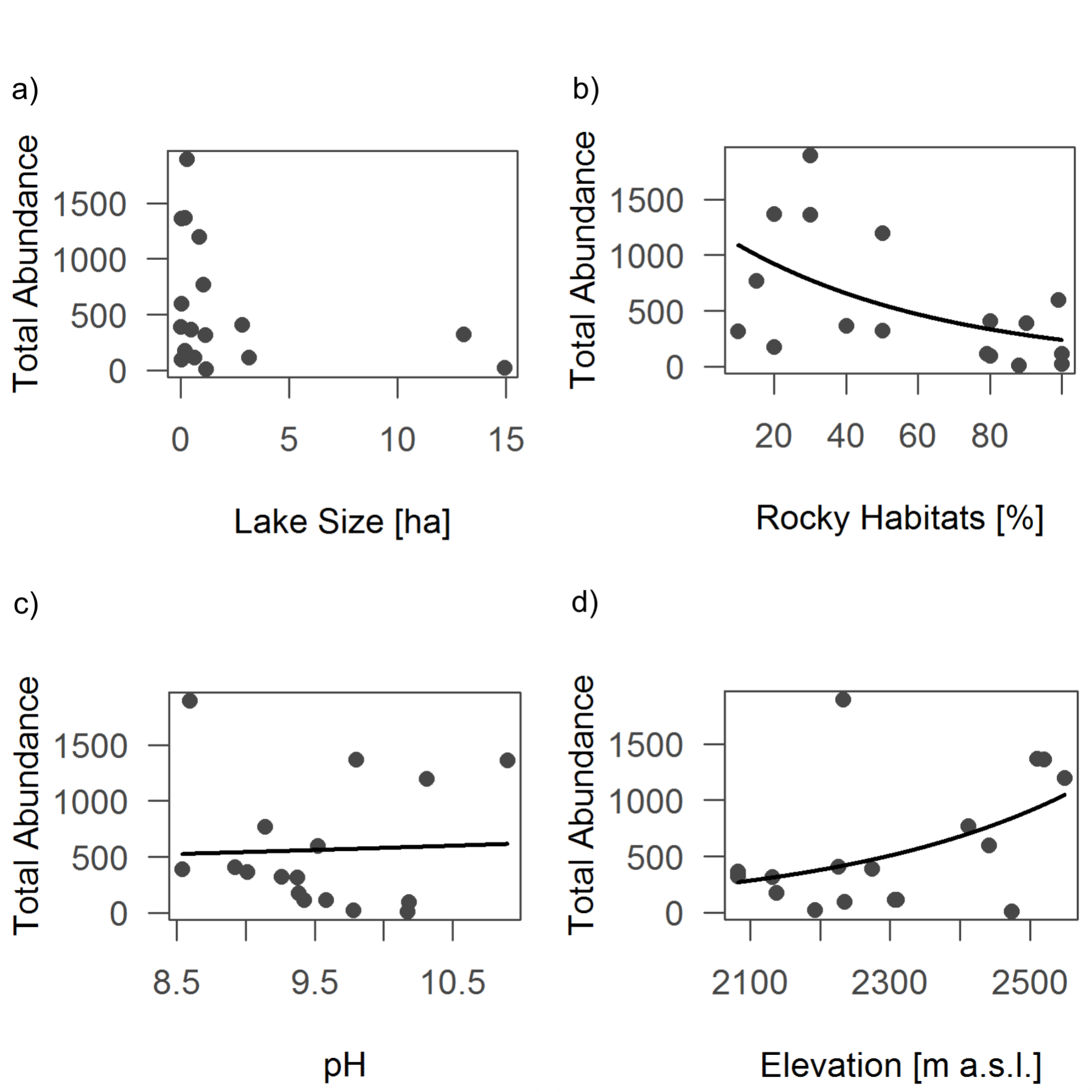

Supplement: S3 Fig — Effect of total macroinvertebrate abundances in a subset of alpine lakes (number 1–17) for which zoo- and phytoplankton abundances per liter were available and were used as additional explanatory variables and a) lake size; b) proportion of rocky habitats (sum of small rocks and sheer rock faces/boulders); c) pH and d) elevation. Regression lines are from generalized linear regression with quasipoisson distributions and log-links and only shown for significant relationships (see S5 Table). (TIF) [file pone.0255619.s003.tif]

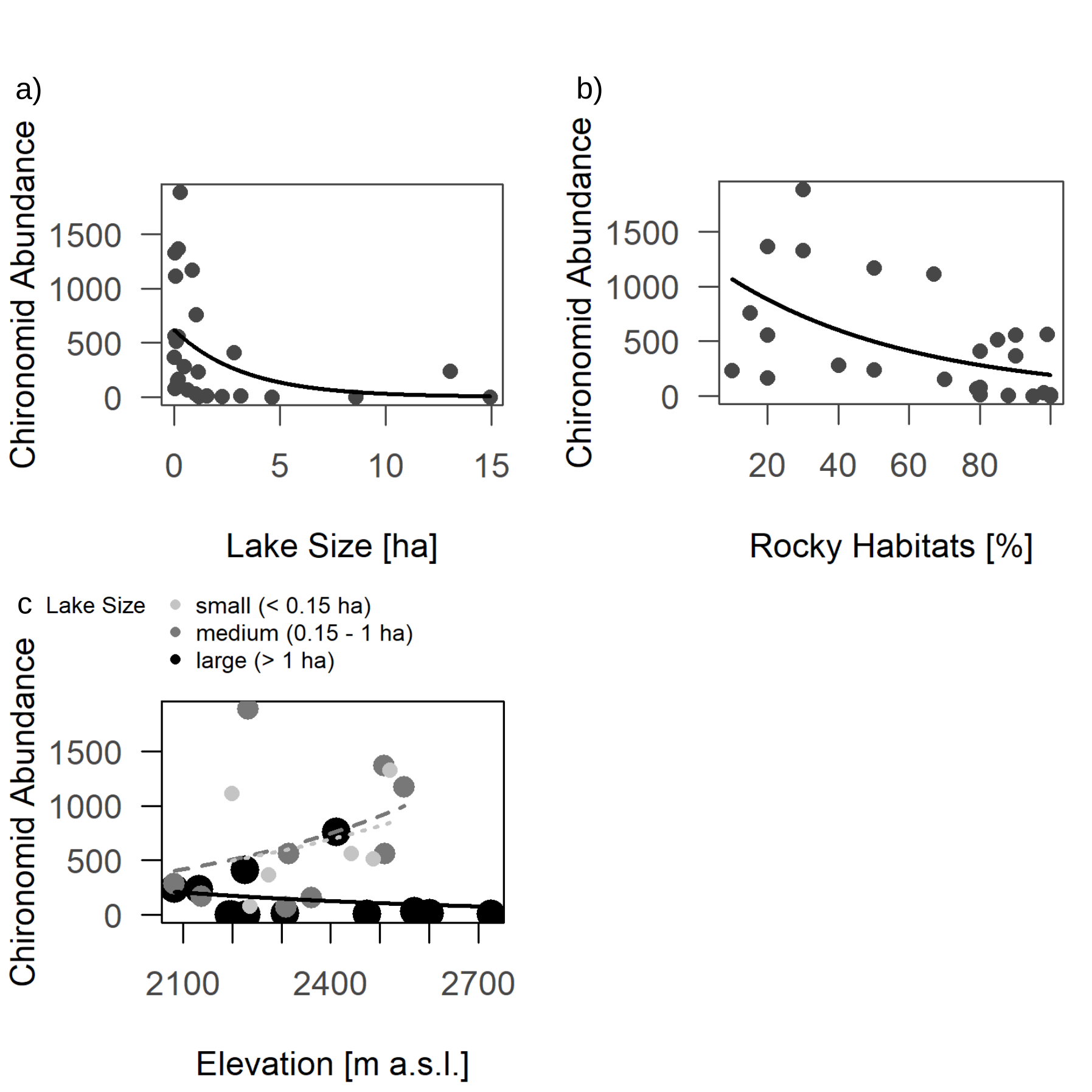

Supplement: S4 Fig — The relationship between chironomid abundances in alpine lakes and a) lake size; b) proportion of rocky habitats (sum of small rocks and sheer rock faces/boulders; and c) the interaction of elevation and lake size, where lake size is visualized by different shades of grey and size of the dots. Regression lines are based on generalized linear regression with a quasipoisson distribution and a log-link and are significant (S7 Table). (TIF) [file pone.0255619.s004.tif]

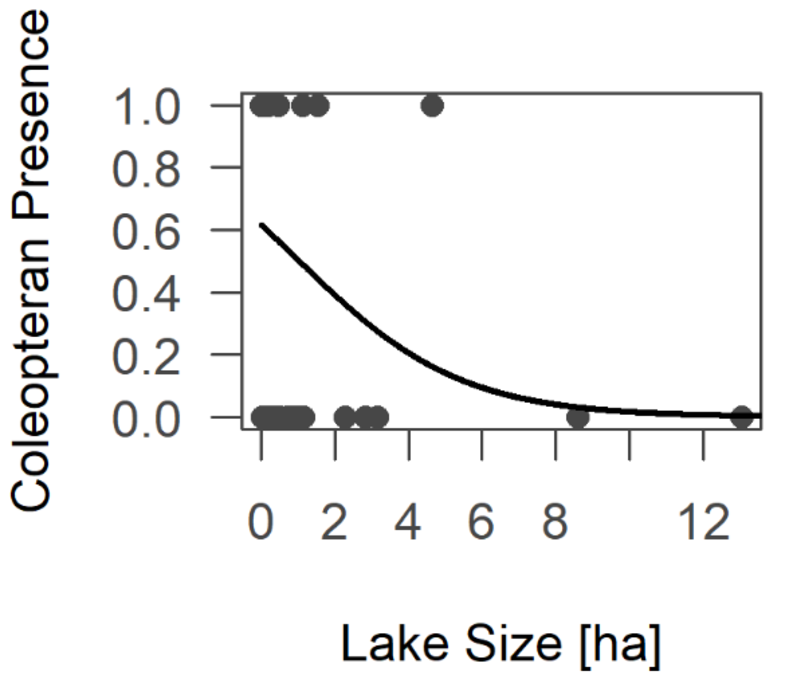

Supplement: S5 Fig — The effect shown here was significant (S7 Table). 1 = coleopterans were present, 0 = coleopterans were absent. (TIF) [file pone.0255619.s005.tif]

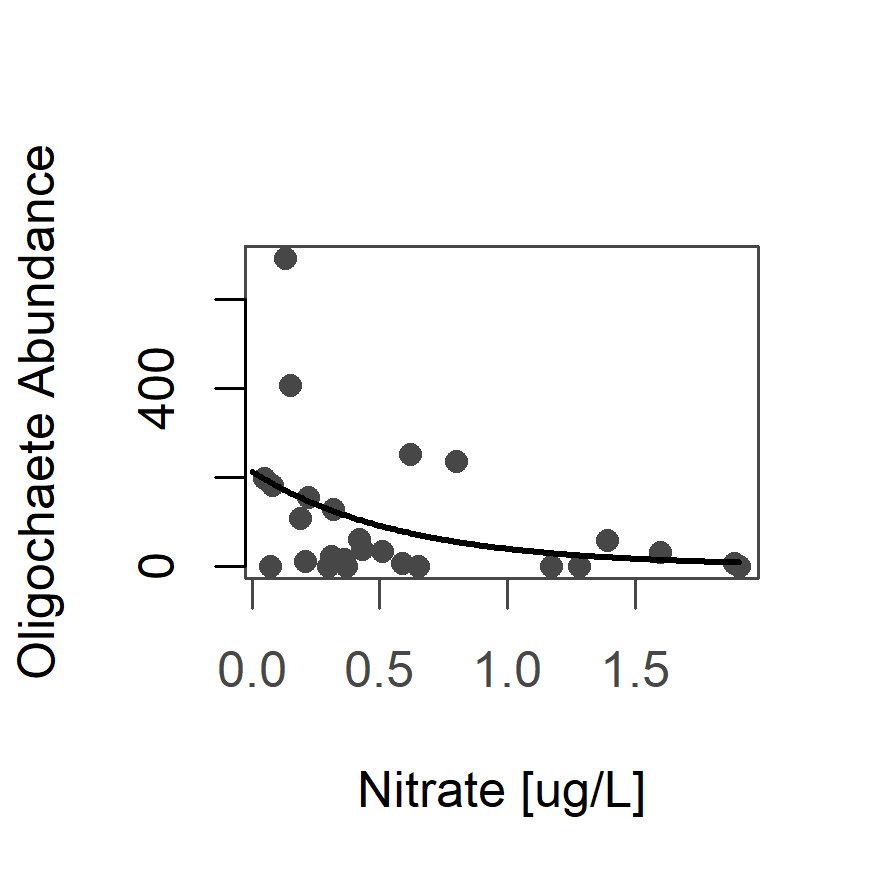

Supplement: S6 Fig — The effect shown here was significant (S7 Table). (TIF) [file pone.0255619.s006.tif]
