# Supplementary material for: Littoral macroinvertebrate communities of alpine lakes along an elevational gradient (Hohe Tauern National Park, Austria)
Source: PLoS One. 2021 Nov 29;16(11):e0255619. doi: 10.1371/journal.pone.0255619 (PMC8629281; doi:10.1371/journal.pone.0255619)
Supplement: S1 Table — Habitat types are sheer rock (large boulders and sheer rock faces), small rocks (up to 20 cm x 15 cm x 5 cm) and sediment. Lakes in quotation marks did not have official names and were named for the convenience of this study by the sampling team. (PDF) [file pone.0255619.s007.pdf]

Number	Lake Name	Valley	Elevation	Sampling Date	Sheer Rock [%]	Small Rocks [%]	Sediment [%]	Lake Size [ha]	Maximum depth [m]	Ice-free days
1	„Innergeschlöß 2“	Innergeschlöß	2,550	31.07.2018	48	2	50	0.86	7.2	66.00
2	„Innergeschlöß 3“	Innergeschlöß	2,520	31.07.2018	10	20	70	0.04	1	NA
3	„Gletscherplateau“	Innergeschlöß	2,235	01.08.2018	60	20	20	0.03	1.25	129.00
4	Salzbodensee	Innergeschlöß	2,138	01.08.2018	19	1	80	0.20	5.1	NA
5	„See nahe Löbbensee“	Innergeschlöß	2,233	02.08.2018	30	0	70	0.30	5.4	73.00
6	Löbbensee	Innergeschlöß	2,226	02.08.2018	70	10	20	2.84	16	129.00
7	Kleiner Tauernsee	Seebachtal	2,310	17.07.2018	79	0	21	0.63	9	67.00
8	Grüneckersee	Seebachtal	2,307	17.07.2018	100	0	0	3.16	20	114.00
9	„Schneefeldsee“	Seebachtal	2,474	18.07.2018	44	44	12	1.17	7.6	55.00
10	„Plattensee“	Seebachtal	2,441	18.07.2018	99	0	1	0.03	1.9	102.00
12	„Großes Elend“	Seebachtal	2,510	19.07.2018	18	2	80	0.19	3.4	98.00
13	„See neben Seebachsee“	Obersulzbachtal	2,083	25.07.2018	20	20	60	0.48	13	108.00
14	Seebachsee	Obersulzbachtal	2,083	25.07.2018	25	25	50	13.05	15	153.00
15	Foisskarsee	Obersulzbachtal	2,132	25.07.2018	10	0	90	1.13	2.7	157.00
16	Sulzsee	Obersulzbachtal	2,193	24.07.2018	10	90	0	14.95	42.4	NA
17	„Obervorderjaidbachsee“	Obersulzbachtal	2,412	26.07.2018	10	5	85	1.05	6	113.00
18	„Untervorderjaidbachsee“	Obersulzbachtal	2,274	26.07.2018	86	4	10	0.02	2	113.00
19	Barrenlesee	Leibnitzbachtal	2,727	06.08.2018	100	0	0	2.27	NA	NA

Number	Lake Name	Valley	Elevation	Sampling Date	Sheer Rock [%]	Small Rocks [%]	Sediment [%]	Lake Size [ha]	Maximum depth [m]	Ice-free days
20	„Kleiner Barrenlesee“	Leibnitzbachtal	2,511	06.08.2018	10	10	80	0.18	NA	NA
21	Gartlesee	Leibnitzbachtal	2,570	07.08.2018	70	28	2	1.01	NA	NA
22	„Leibnitzkopfpfütze“	Leibnitzbachtal	2,650	07.08.2018	50	30	20	0.02	NA	NA
23	„Debantsee“	Leibnitzbachtal	2,486	07.08.2018	10	75	15	0.10	NA	NA
24	Schwarzseele	Dorfer Tal	2,601	08.08.2018	30	50	20	1.54	NA	NA
26	„Murmelblubber“	Dorfer Tal	2,316	08.08.2018	30	60	10	0.18	NA	NA
27	„Elisabethsee“	Felbertal	2,361	09.08.2018	30	40	30	0.17	NA	NA
28	Plattachsee	Felbertal	2,199	10.08.2018	70	30	0	8.59	NA	NA
29	„Kleiner Plattachsee“	Felbertal	2,199	10.08.2018	33	34	33	0.07	NA	NA
30	Langsee	Felbertal	2,229	10.08.2018	35	60	5	4.63	NA	NA
