# Supplementary material for: Littoral macroinvertebrate communities of alpine lakes along an elevational gradient (Hohe Tauern National Park, Austria)
Source: PLoS One. 2021 Nov 29;16(11):e0255619. doi: 10.1371/journal.pone.0255619 (PMC8629281; doi:10.1371/journal.pone.0255619)
Supplement: S2 Table — (PDF) [file pone.0255619.s008.pdf]

Number	Name	Temperature [°C]	Dissolved oxygen [%]	Conductivity [µS/cm]	pH	Nitrate [mg/L]	turbidity [FNU]	Blue-green algae pycocyanin [µg/l]	Chlorophyll-a [µg/l]	Zooplankton abundance / L	Phytoplankton abundance / L
1	“Innergeschlöss 2”	9.24	82.25	24.35	10.31	0.21	0.49	0.40	0.77	25.84	0
2	“Innergeschlöss 3”	13.07	72.30	24.40	10.90	0.15	0.40	0.39	1.15	0.07	23000
3	“Gletscherplateau”	17.09	83.85	45.35	10.18	0.51	1.96	0.57	10.28	14.03	2436700
4	Salzbodensee	17.99	80.20	10.85	9.38	0.05	0.53	0.31	1.19	2.71	4762800
5	“See nahe Loebbensee”	11.33	76.25	59.30	8.59	0.32	0.79	0.36	0.69	0.02	17200
6	Loebbensee	12.70	80.70	47.95	8.92	0.13	0.41	0.42	0.75	225.59	0
7	Kleiner Tauernsee	4.84	77.80	15.50	9.42	1.89	0.30	0.38	0.58	0.91	0
8	Grueneckersee	6.68	73.96	12.30	9.58	0.37	1.06	0.34	0.61	5.04	0
9	“Schneefeldsee”	2.41	73.15	19.00	10.17	1.28	2.17	0.46	0.81	0.02	0
10	“Plattensee”	8.44	72.45	10.35	9.52	0.43	0.35	0.44	1.12	6.87	0
12	“Großes Elend”	5.26	74.20	14.35	9.80	0.80	0.26	0.29	0.29	0.07	0
13	“See neben Seebachsee”	8.75	81.15	27.60	9.01	0.36	0.36	0.40	0.94	31.48	341600
14	Seebachsee	11.39	81.90	40.60	9.26	0.19	0.94	0.28	0.70	4.20	304100
15	Foisskarsee	11.59	81.63	26.50	9.37	0.22	0.19	0.33	0.43	0.01	0
16	Sulzsee	4.53	76.65	11.85	9.78	1.91	41.22	0.57	0.96	0.00	0
17	“Obervorderjaidbachsee”	6.22	73.60	13.25	9.14	0.65	2.97	0.37	0.58	0.01	0
18	“Untervorderjaidbachsee”	14.22	80.65	2.60	8.54	0.07	0.97	0.22	1.87	25.74	539600
19	Barrenlesee	10.13	75.45	17.30	9.63	1.17	0.43	0.38	0.81	NA	NA
20	“Kleiner Barrenlesee”	9.90	88.90	116.80	9.30	1.60	0.56	0.41	0.81	NA	NA
21	Gartlesee	13.20	80.10	81.90	8.81	0.51	0.36	0.39	0.76	NA	NA
22	“Leibnitzkopfpfütze”	10.35	69.20	NA	NA	NA	NA	NA	NA	NA	NA

Number	Name	Temperature [°C]	Dissolved oxygen [%]	Conductivity [µS/cm]	pH	Nitrate [mg/L]	turbidity [FNU]	Blue-green algae pycocyanin [µg/l]	Chlorophyll-a [µg/l]	Zooplankton abundance / L	Phytoplankton abundance / L
23	"Debantsee"	12.62	81.30	150.30	8.05	1.39	0.91	0.45	0.95	NA	NA
24	Schwarzseele	13.80	81.45	78.20	8.98	0.62	0.69	0.31	0.95	NA	NA
26	"Murmelblubber"	15.31	83.00	16.90	8.41	0.08	0.43	0.16	0.32	NA	NA
27	"Elisabethsee"	14.84	81.90	44.25	8.72	0.42	1.26	0.11	0.37	NA	NA
28	Plattachsee	11.27	79.20	26.10	8.54	0.30	0.51	0.22	0.97	NA	NA
29	"Kleiner Plattachsee"	12.83	68.80	5.00	8.14	0.31	1.09	0.22	1.50	NA	NA
30	Langsee	12.51	74.40	20.50	7.99	0.59	0.37	0.17	0.82	NA	NA

S 3 Table. List of all orders or subclasses, the lowest taxon they were determined to, total abundances across the study, the number of sampling sites (lakes) they were present in and the number of taxa within each order/subclass that were determined for macroinvertebrates in alpine lakes of Hohe Tauern National Park.

Order / Subclass	lowest taxon determined	Total Abundance	# Sampling Sites	# Taxa determined
Coleoptera (adult)	Species	42	7	5
Coleoptera (juvenile)	Genus	155	12	5
Diptera	Family	11,925	25	6
Hemiptera	Genus	9	2	1
Plecoptera	Species	23	5	3
Trichoptera	Family	534	21	1
Tricladida	Species	22	5	1
Trombidiformes	Family	44	2	1
Veneroida	Genus	142	4	1
Oligochaeta	Subclass	5,080	22	1
Hirudinea	Subclass	1	1	1

S 4 Table. Table of correlations between measured variables in the lakes of Hohe Tauern National Park according to Pearson's product-moment correlation test.

Parameter 1	Parameter 2	P	r
Conductivity	**Dissolved Oxygen**	0.004	0.537
Temperature	**Dissolved Oxygen**	0.031	0.416
Temperature	**Nitrate**	0.001	-0.586
Turbidity	**Nitrate**	0.013	0.473
Cyanobacteria	**Nitrate**	0.013	0.472
Cyanobacteria	Turbidity	0.035	0.408
Cyanobacteria	**pH**	0.002	0.566
Cyanobacteria	**Chlorophyll-a**	0.040	0.398
GPS E	**Elevation**	0.018	0.452
GPS E	**Temperature**	0.010	-0.486
GPS E	GPS N	<0.001	-0.635
GPS E	**Nitrate**	0.036	0.405
GPS N	**Elevation**	<0.001	-0.714
GPS N	Conductivity	0.007	-0.509
GPS N	**Nitrate**	0.003	-0.552

This correlation test was the basis for choosing parameters for further statistical analysis. Only significant correlations are listed. Parameters printed in bold were included in further statistical analysis.

S 5 Table. Results of generalized linear modelling (GLM), applying quasipoisson distribution and a log link for total abundance and family richness in alpine lakes of Hohe Tauern National Park (Austria) for a subset of lakes (lakes number 1-17) for which zoo- and phytoplankton abundances per liter were available and were used as additional explanatory variables.

	Total Abundance		*Family Richness*	
	F	P	F	P
Elevation	8.58	**0.025**	0.07	0.799
Lake Size	2.42	0.171	0.22	0.654
Rocky Habitats	9.92	**0.020**	5.28	0.061
Habi. Div.	0.02	0.881	2.08	0.199
Dis. Oxygen	0.03	0.871	0.33	0.586
Nitrate	4.10	0.089	0.27	0.621
Abundance Phytoplankton / L	1.85	0.084	0.11	0.749
Abundance Zooplankton / L	0.30	0.963	1.60	0.252

pH	4.66	0.074	0.90	0.379
Elevation:Area	1.49	0.268	0.12	0.747

Numerator df=1 for each explanatory variable, significant P-values are printed in bold. Residual degrees of freedom: 16.

S 6 Table. Results of generalized linear modelling (GLM), applying quasipoisson distribution and a log link for Chironomidae abundance and Oligochaeta abundance and applying binomial distribution and a logit link for Coleopteran presence in alpine lakes of "Hohe Tauern" National Park (Austria).

	Chironomidae		Coleoptera		Oligochaeta	
	F	P	F	P	F	P
Elevation	0.90	0.355	36.20	0.275	0.03	0.870
Lake Size	8.48	**0.010**	27.60	**0.003**	0.04	0.835
Rocky Habitats	12.71	**0.002**	26.17	0.231	1.44	0.247
Habi. Div.	1.466	0.243	25.09	0.300	1.69	0.212
Dis. Oxygen	4.31	0.053	21.82	0.070	0.04	0.850
Nitrate	2.75	0.116	21.66	0.694	4.71	**0.045**
Chlorophyll-a	0.75	0.399	17.85	0.051	0.56	0.465
pH	2.54	0.129	15.13	0.100	0.11	0.747
Elevation:Lake Size	8.40	**0.0100**	15.07	0.800	0.15	0.704

Numerator df=1 for each explanatory variable, significant P-values are printed in bold. Residual degrees of freedom: 26

S 7 Table. Results of generalized linear modelling (GLM), applying quasipoisson distribution and a log link for trichopteran abundances in alpine lakes of Hohe Tauern National Park (Austria).

	Trichoptera	
	F	P
Elevation	0.01	0.932
Lake Size	1.02	0.326
Rocky Habitats	3.76	0.069
Habi. Div.	0.20	0.662
Dis. Oxygen	0.27	0.609
Nitrate	0.49	0.495
Chlorophyll-a	0.54	0.471

pH	0.28	0.607
Elevation:Lake Size	0.05	0.821

Numerator df=1 for each explanatory variable, significant P-values are printed in bold. Residual degrees of freedom: 26

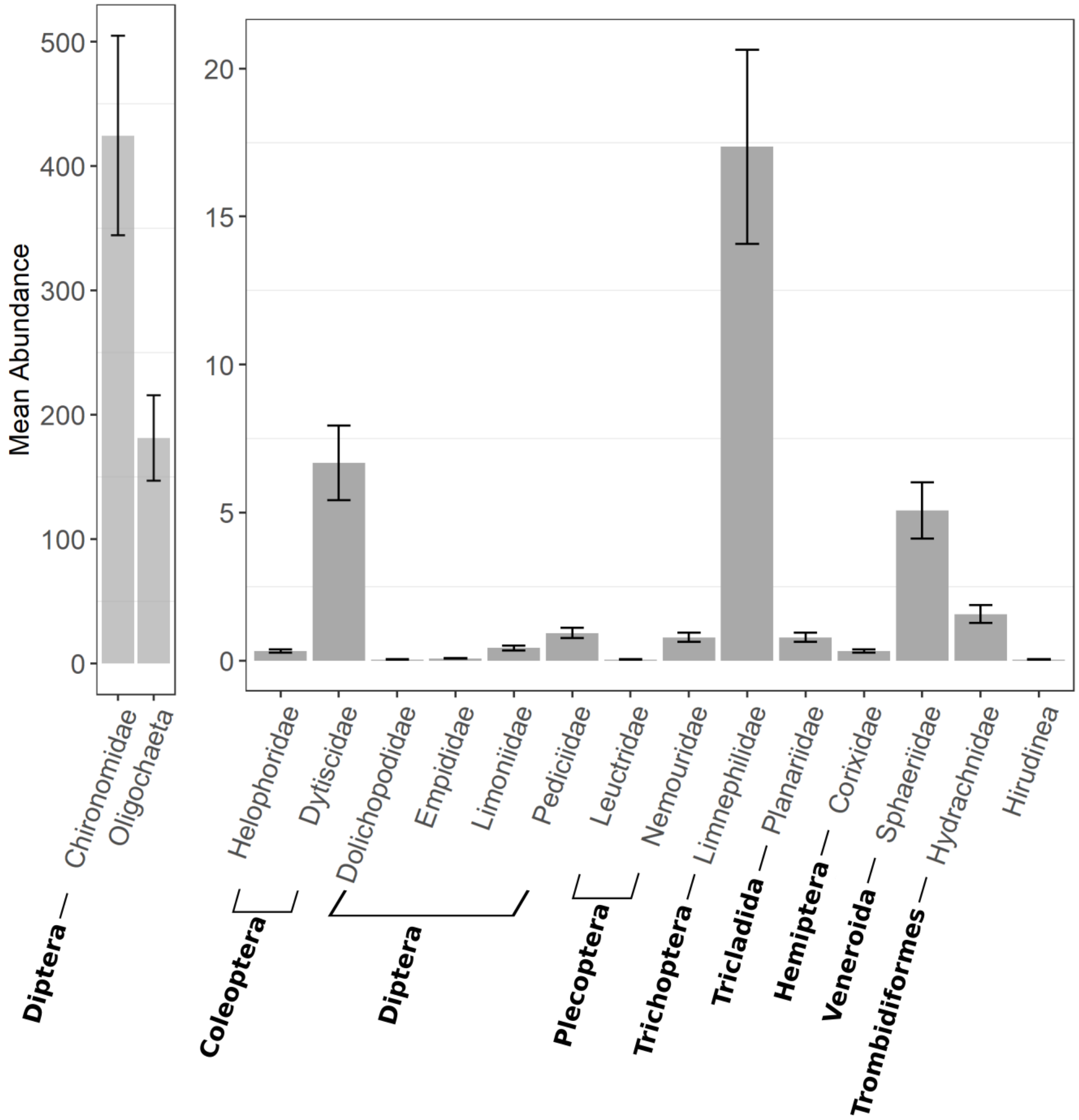

S 1 Fig. Mean abundances of families/subclasses across the study of macroinvertebrates in alpine lakes of Hohe Tauern National Park. Standard error is given by error bars. Due to large differences in abundance, chironomids and oligochaetes are displayed on a different scale.

36

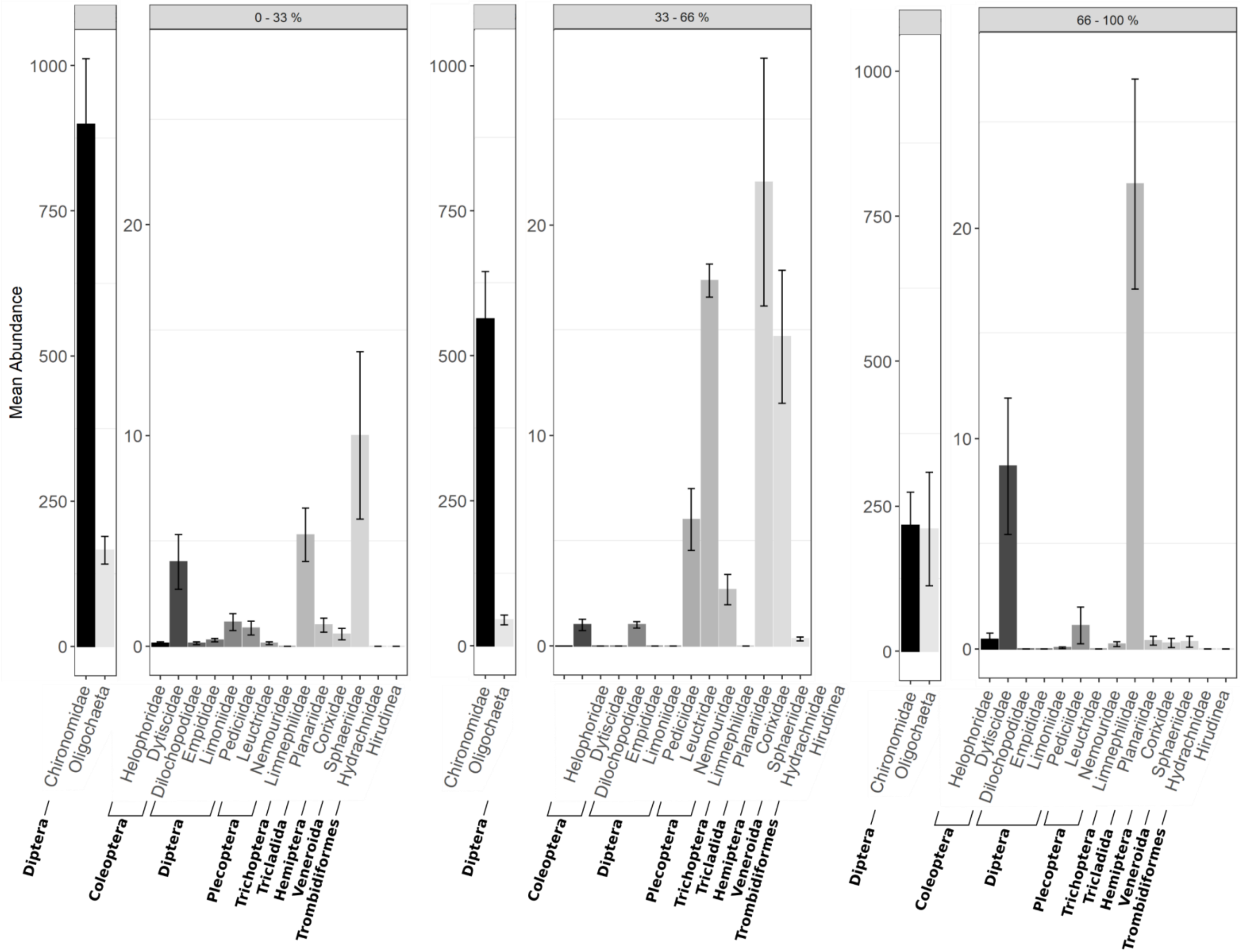

S 2 Fig. Mean abundances of families/subclasses across the study of macroinvertebrates in alpine lakes of Hohe Tauern National Park, grouped by share of rocky habitat (given in percent on top of graphs). Standard error is given by error bars. Due to large differences in abundance, chironomids and oligochaetes are displayed on a different scale. Oder names are given below taxa.

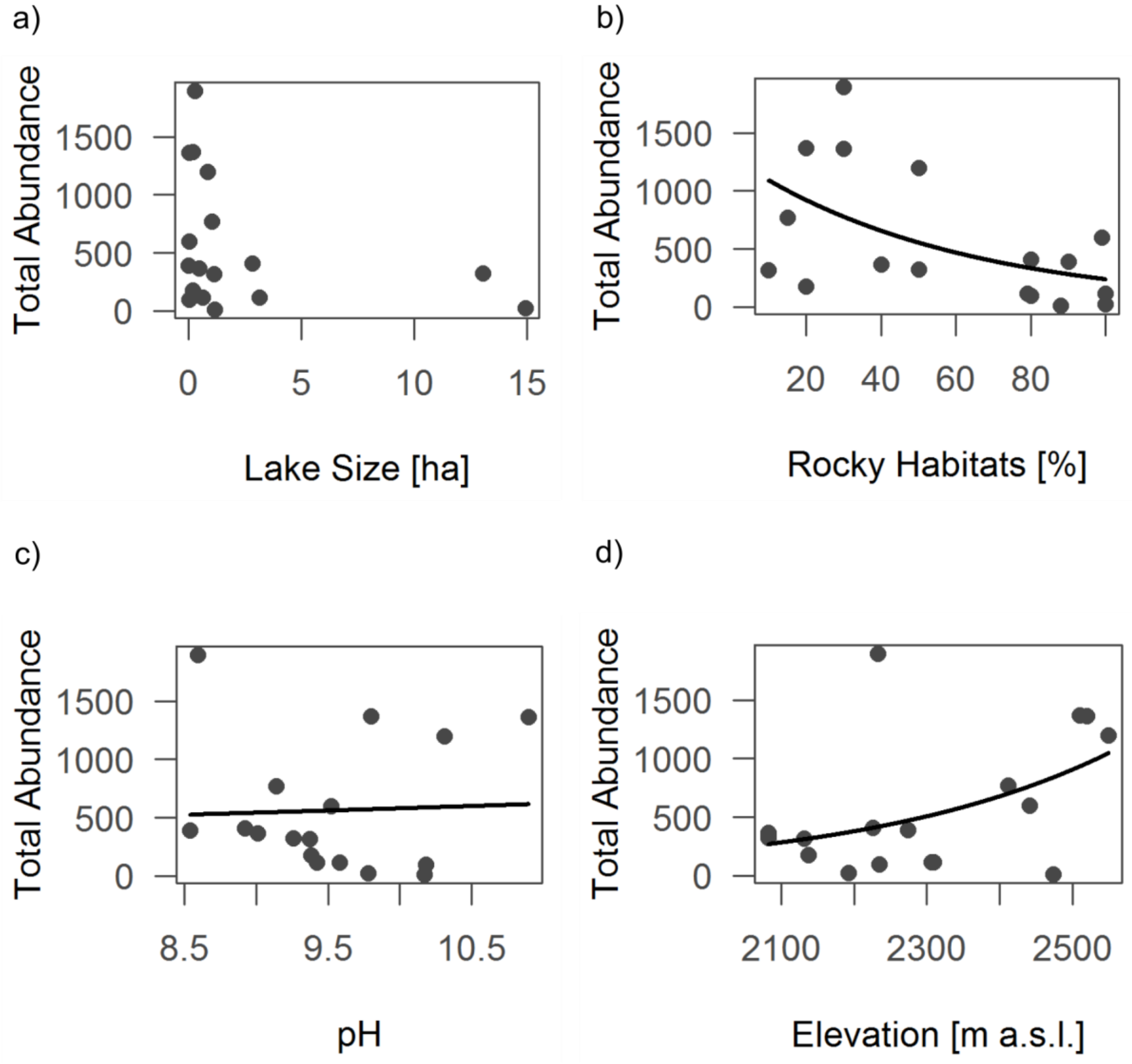

S 3 Fig. Effect of total macroinvertebrate abundances in a subset of alpine lakes (number 1-17) for which zoo- and phytoplankton abundances per liter were available and were used as additional explanatory variables and a) lake size; b) proportion of rocky habitats (sum of small rocks and sheer rock faces / boulders); c) pH and d) elevation. Regression lines are from generalized linear regression with quasipoisson distributions and log-links and only shown for significant relationships (see S 5 Table).

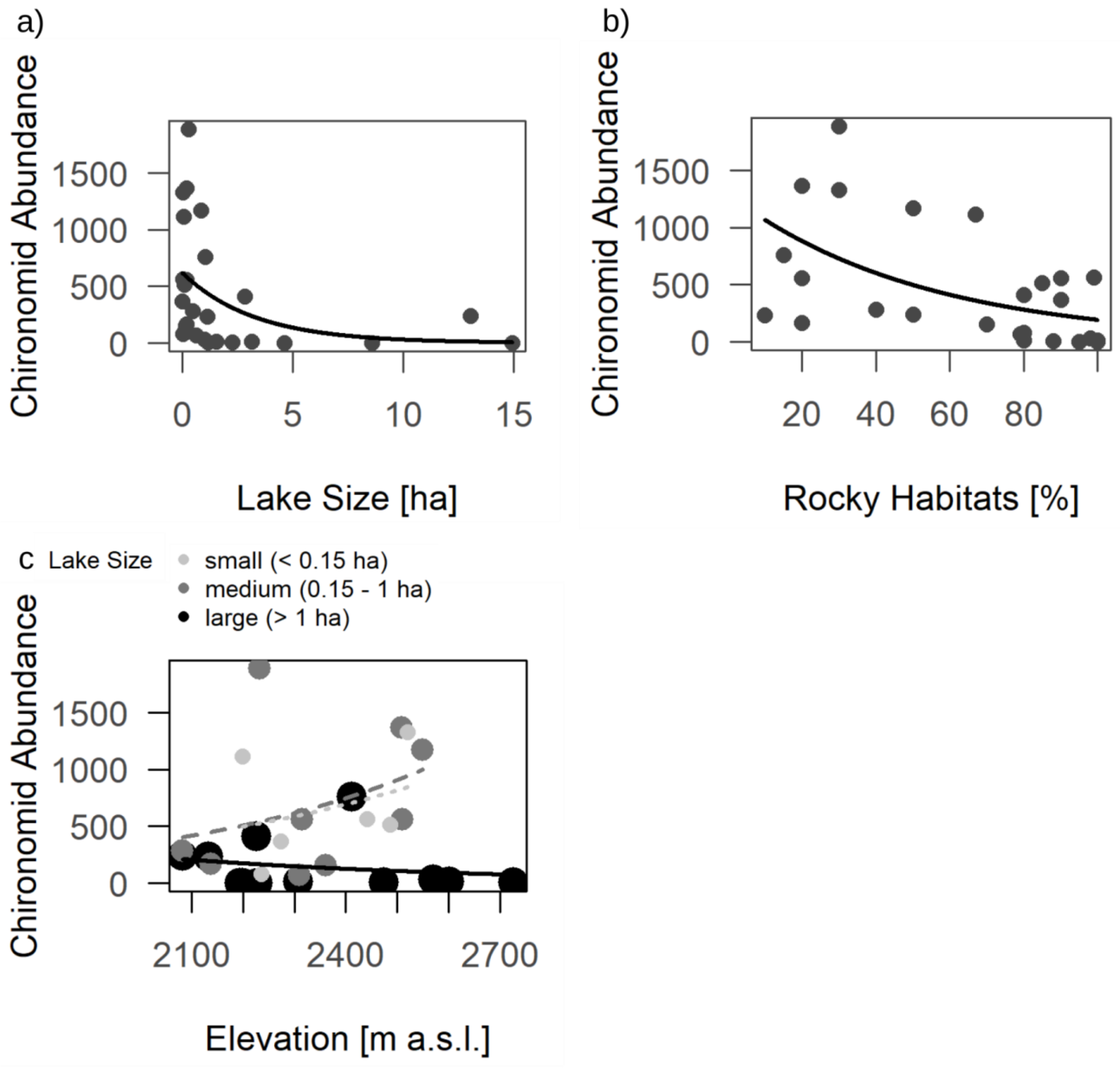

S 4 Fig. The relationship between chironomid abundances in alpine lakes and a) lake size; b) proportion of rocky habitats (sum of small rocks and sheer rock faces / boulders; and c) the interaction of elevation and lake size, where lake size is visualized by different shades of grey and size of the dots. Regression lines are based on generalized linear regression with a quasipoisson distribution and a log-link and are significant (S 7 Table).

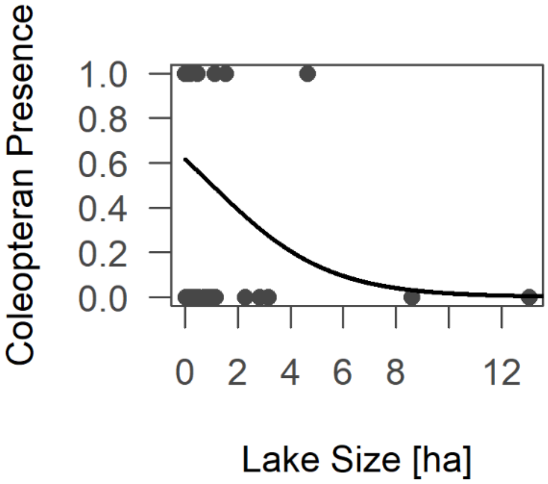

S 5 Fig. Generalized linear regression with binomial distribution and logit-link between coleopteran presence in alpine lakes and lake size. The effect shown here was significant (S 7 Table). 1 = coleopterans were present, 0 = coleopterans were absent.

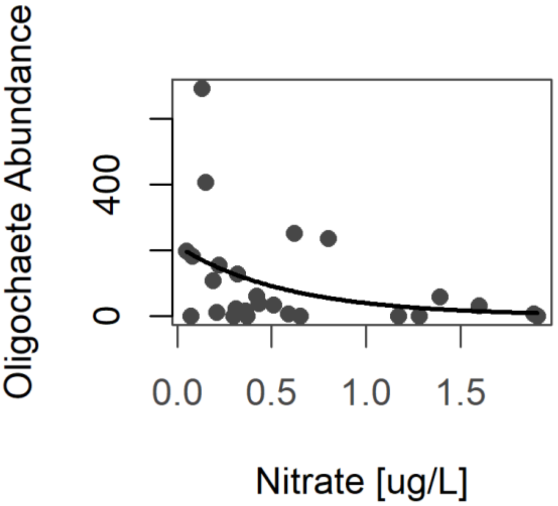

S 6 Fig. Generalized linear regression with quasipoisson distribution and log-link between oligochaete abundances and nitrate in alpine lakes. The effect shown here was significant (S 7 Table).
