# Supplementary material for: Littoral macroinvertebrate communities of alpine lakes along an elevational gradient (Hohe Tauern National Park, Austria)
Source: PLoS One. 2021 Nov 29;16(11):e0255619. doi: 10.1371/journal.pone.0255619 (PMC8629281; doi:10.1371/journal.pone.0255619)
Supplement: S3 Table — (PDF) [file pone.0255619.s009.pdf]

Order / Subclass	lowest taxon determined	Total Abundance	# Sampling Sites	# Taxa determined
Coleoptera (adult)	Species	42	7	5
Coleoptera (juvenile)	Genus	155	12	5
Diptera	Family	11,925	25	6
Hemiptera	Genus	9	2	1
Plecoptera	Species	23	5	3
Trichoptera	Family	534	21	1
Tricladida	Species	22	5	1
Trombidiformes	Family	44	2	1
Veneroida	Genus	142	4	1
Oligochaeta	Subclass	5,080	22	1
Hirudinea	Subclass	1	1	1
