# Supplementary material for: Littoral macroinvertebrate communities of alpine lakes along an elevational gradient (Hohe Tauern National Park, Austria)
Source: PLoS One. 2021 Nov 29;16(11):e0255619. doi: 10.1371/journal.pone.0255619 (PMC8629281; doi:10.1371/journal.pone.0255619)
Supplement: S4 Table — This correlation test was the basis for choosing parameters for further statistical analysis. Only significant correlations are listed. Parameters printed in bold were included in further statistical analysis. (PDF) [file pone.0255619.s010.pdf]

Parameter 1	Parameter 2	P	r
Conductivity	**Dissolved Oxygen**	0.004	0.537
Temperature	**Dissolved Oxygen**	0.031	0.416
Temperature	**Nitrate**	0.001	-0.586
Turbidity	**Nitrate**	0.013	0.473
Cyanobacteria	**Nitrate**	0.013	0.472
Cyanobacteria	Turbidity	0.035	0.408
Cyanobacteria	**PH**	0.002	0.566
Cyanobacteria	**Chlorophyll-a**	0.040	0.398
GPS E	**Elevation**	0.018	0.452
GPS E	**Temperature**	0.010	-0.486
GPS E	GPS N	<0.001	-0.635
GPS E	**Nitrate**	0.036	0.405
GPS N	**Elevation**	<0.001	-0.714
GPS N	Conductivity	0.007	-0.509
GPS N	**Nitrate**	0.003	-0.552
