# Supplementary material for: Littoral macroinvertebrate communities of alpine lakes along an elevational gradient (Hohe Tauern National Park, Austria)
Source: PLoS One. 2021 Nov 29;16(11):e0255619. doi: 10.1371/journal.pone.0255619 (PMC8629281; doi:10.1371/journal.pone.0255619)
Supplement: S5 Table — Numerator df = 1 for each explanatory variable, significant P-values are printed in bold. Residual degrees of freedom: 16. (PDF) [file pone.0255619.s011.pdf]

	Total Abundance		*Family Richness*	
	F	P	F	P
Elevation	8.58	**0.025**	0.07	0.799
Lake Size	2.42	0.171	0.22	0.654
Rocky Habitats	9.92	**0.020**	5.28	0.061
Habi. Div.	0.02	0.881	2.08	0.199
Dis. Oxygen	0.03	0.871	0.33	0.586
Nitrate	4.10	0.089	0.27	0.621
Abundance Phytoplankton / L	1.85	0.084	0.11	0.749
Abundance Zooplankton / L	0.30	0.963	1.60	0.252
pH	4.66	0.074	0.90	0.379
Elevation:Area	1.49	0.268	0.12	0.747
