# Supplementary material for: Littoral macroinvertebrate communities of alpine lakes along an elevational gradient (Hohe Tauern National Park, Austria)
Source: PLoS One. 2021 Nov 29;16(11):e0255619. doi: 10.1371/journal.pone.0255619 (PMC8629281; doi:10.1371/journal.pone.0255619)
Supplement: S6 Table — Numerator df = 1 for each explanatory variable, significant P-values are printed in bold. Residual degrees of freedom: 26. (PDF) [file pone.0255619.s012.pdf]

	Trichoptera	
	F	P
Elevation	0.01	0.932
Lake Size	1.02	0.326
Rocky Habitats	3.76	0.069
Habi. Div.	0.20	0.662
Dis. Oxygen	0.27	0.609
Nitrate	0.49	0.495
Chlorophyll-a	0.54	0.471
pH	0.28	0.607
Elevation:Lake Size	0.05	0.821
