# Supplementary material for: Littoral macroinvertebrate communities of alpine lakes along an elevational gradient (Hohe Tauern National Park, Austria)
Source: PLoS One. 2021 Nov 29;16(11):e0255619. doi: 10.1371/journal.pone.0255619 (PMC8629281; doi:10.1371/journal.pone.0255619)
Supplement: S7 Table — Numerator df = 1 for each explanatory variable, significant P-values are printed in bold. Residual degrees of freedom: 26. (PDF) [file pone.0255619.s013.pdf]

	Chironomidae		Coleoptera		Oligochaeta	
	F	P	F	P	F	P
Elevation	0.90	0.355	36.20	0.275	0.03	0.870
Lake Size	8.48	**0.010**	27.60	**0.003**	0.04	0.835
Rocky Habitats	12.71	**0.002**	26.17	0.231	1.44	0.247
Habi. Div.	1.466	0.243	25.09	0.300	1.69	0.212
Dis. Oxygen	4.31	0.053	21.82	0.070	0.04	0.850
Nitrate	2.75	0.116	21.66	0.694	4.71	**0.045**
Chlorophyll-a	0.75	0.399	17.85	0.051	0.56	0.465
pH	2.54	0.129	15.13	0.100	0.11	0.747
Elevation:Lake Size	8.40	**0.0100**	15.07	0.800	0.15	0.704
